# Growth, secondary metabolite production, and in vitro antiplasmodial activity of *Sonchus arvensis* L. callus under dolomite [CaMg(CO_3_)_2_] treatment

**DOI:** 10.1371/journal.pone.0254804

**Published:** 2021-08-20

**Authors:** Dwi Kusuma Wahyuni, Shilfiana Rahayu, Andi Hamim Zaidan, Wiwied Ekasari, Sehanat Prasongsuk, Hery Purnobasuki

**Affiliations:** 1 Department of Biology, Faculty of Science and Technology, Universitas Airlangga, Surabaya, East Java, Indonesia; 2 Plant Biomass Utilization Research Unit, Department of Botany, Faculty of Science, Chulalongkorn University, Bangkok, Thailand; 3 Biotechnology of Tropical Medicinal Plants Research Group, Universitas Airlangga, Surabaya, East Java, Indonesia; 4 Department of Biology, Faculty of Science and Technology, Universitas Islam Negeri Sunan Kalijaga, Yogyakarta, Indonesia; 5 Department of Physics, Faculty of Science and Technology, Universitas Airlangga, Surabaya, East Java, Indonesia; 6 Department of Pharmaceutical Sciences, Faculty of Pharmacy, Universitas Airlangga, Surabaya, East Java, Indonesia; National University of Kaohsiung, TAIWAN

## Abstract

Malaria is still a global health problem. *Plasmodium* is a single-cell protozoan parasite that causes malaria and is transmitted to humans through the female *Anopheles* mosquito. The previous study showed that *Sonchus arvensis* L. callus has antiplasmodial activity. Several treatments are needed for callus quality improvement for antimalarial compound production. This study aimed to examine the effect of dolomite [CaMg(CO_3_)_2_] on growth (morpho-anatomical structure and biomass), secondary metabolite production, and *in vitro* antiplasmodial activity of *S*. *arvensis* L. callus. In this study, leaf explants were grown in Murashige and Skoog medium with a combination of 2,4-dichlorophenoxyacetic acid (2,4-D, one mg/L) and 6-benzyl amino purine (BAP, 0.5 mg/L) with dolomite (50, 75, 100, 150, and 200 mg/L). The 21 days callus ethanolic and methanolic extract were analyzed by gas chromatography-mass spectrometry (GC-MS) and thin-layer chromatography (TLC). The antiplasmodial test was performed on a blood culture infected with *Plasmodium falciparum* strain 3D7 using the Rieckmann method. The results showed that dolomite significantly affected callus growth, metabolite profile, and in vitro antiplasmodial activity. Dolomite (150 mg/L) showed the highest biomass (0.590 ± 0.136 g fresh weight and 0.074 ± 0.008 g dry weight). GC-MS analysis detected four compounds from callus ethanolic extract. Pelargonic acid, decanoic acid, and hexadecanoic acid were major compounds. One new terpenoid compound is based on TLC analysis. *S*. *arvensis* L. callus has antiplasmodial activity with the IC_50_ value of 5.037 μg/mL. It was three times lower than leaf methanolic extract and five times lower than leaf ethanolic extract.

## Introduction

Malaria is well known as the world’s primary disease, especially in the tropics. It is endemic in Asian, African, and Latin American countries [1]. Millennium development goals target to stop the spread and decrease malaria incidence through indicators of reducing morbidity and mortality caused by malaria [2]. Malaria prevention efforts are significant, including malaria prevention, diagnosis, and treatment. Eighty percent of the world population still uses natural products for medicine, and 75% of patients with malaria use traditional medication to treat this disease [3]. Previously, malaria medicine was derived from *Chincona succirubra* L., while a new malaria drug generation, artemisinin, was made from *Artemisia annua* L. [4].

*Sonchus arvensis* L., an annual herb that belongs to Asteraceae, has an erect habit that reaches 64 cm in height [5]. In Indonesia, it is the 7^th^ most popular medicinal plant [6]. Flavonoids, coumarin, taraxasterol, phenolic acids, ascorbic acid, and terpenoids are detected in this plant [7]. Many studies show that *S*. *arvensis* L. possesses antioxidant activity, uric acid-lowering activity, anti-inflammatory activity [8], immunomodulatory activity [9], and antibacterial activity [10]. In addition, a previous report showed that *S*. *arvensis* L. callus has antimalarial activity [11].

Due to there is no cultivation of this plant, it is challenging to produce *S*. *arvensis* L. raw material and its medicinal properties to meet the market demand. Therefore, for sustainable progress and consumption of *S*. *arvensis* L., it is essential to find methods to solve them. Plant tissue culture can continuously produce secondary metabolites with high economic value relatively quickly, with more consistent and controlled quality and higher content than direct harvest [12].

The factors that influence its success are basic medium [13], growth regulators [14], elicitors [15], and plant genotypes [16]. Dolomite [CaMg(CO_3_)_2_] is a limestone consisting of calcium carbonate and magnesium carbonate, which is widely applied in agriculture to reduce soil acidity and provide nutrients for plants [17]. Dolomite is double salt composed of calcium carbonate (CaCO_3_) and magnesium carbonate (MgCO_3_). The heat treatment of CaCO_3_ and MgCO_3_ respectively generates calcium oxide (CaO) and magnesium oxide (MgO), which have antimicrobial activity [18] and antiviral (H3N3 influenza virus) [19]. In plant growth and development, calcium plays a vital role in cell membranes’ structure, while magnesium is an essential factor that makes up the chlorophyll [20]. Furthermore, the use of dolomite is expected to increase callus growth and its antiplasmodial activity.

In the present study, we evaluated the morpho-anatomy, growth, metabolite profiles, and antiplasmodial activity of *S*. *arvensis* L. callus. This study provides valuable information about the effects of dolomite on the growth and metabolic profiles of *S*. *arvensis* L. callus. It could also improve the mass production of *S*. *arvensis* L. in both agricultural and industrial settings in the future.

## Materials and methods

### Plant material

The plant material was collected from Merapi Mountain slope, Daerah Istimewa Yogyakarta, Indonesia. It was identified by a botanist of Purwodadi Botanical Garden Herbarium, Indonesia Science Institute. Explants were taken from the second and third leaves of the 2-3-month-old plant before the generative stage.

### Dolomite stock preparation

The dolomite [CaMg(CO_3_)_2_] mined in Sekapuk village, Gresik, Indonesia, was used in this study. The dolomite in Sekapuk village contains MgO (18%–22%) and CaO (28%–30%). First, the dolomite stock was made by diluting powder with HCl. Making a dolomite stock of 10,000 mg/L was done by measuring 1 g of dolomite, then dissolving it with HCl and stirring until it was transparent (dissolved well). Finally, distilled water is added until the stock volume reaches 100 mL.

### Media preparation

The callus culture medium used was Murashige and Skoog (MS) medium [21]. MS media with 3% (w/v) sucrose, 8 g/L agar, 2,4-dichlorophenoxyacetic acid (2,4-D; 0, 0.5, 1 mg/L), and 6-benzyl amino purine (BAP; 0, 0.5, 1 mg/L) were used for callus induction. MS medium with 3% (w/v) sucrose, 8 g/L agar, 1 mg/L 2,4-D, 0.5 mg/L BAP, and dolomite (0, 50, 75, 100, 150, and 200 mg/L) were used for callus subculture. The pH of callus induction and dolomite media was adjusted at a range of 5.7–6.3 with 1 N potassium hydroxide (KOH) or 1 N hydrochloric acid (HCl). The media were sterilized in an autoclave at 121°C and 1 atm of pressure for 15 min.

### Explant sterilization, callus induction, and subculture on dolomite media

The second and third leaves were washed with detergent and rinsed with water thrice. The cut leaves were soaked in 40 g/L dithane fungicides for 15 min and then rinsed with sterilized water thrice. The sterilization process continues in the laminar airflow (LAF) cabinet. The leaves are soaked with 15% (v/v) bayclean (activated with 5% sodium hypochlorite) for 15 min, rinsed with sterilized distilled water thrice, then soaked in 70% (v/v) alcohol for 5 s, and rinsed again with sterilized distilled water thrice. The leaves were cut into 1 cm^2^, planted in a culture bottle (100 mL, 30 mL medium), and covered with aluminum foil. The culture was incubated for 31 days at 23°C ± 3°C with 24 h lighting. The light was equipped with continuous (24 h) light of neon lamp (general electric cool white fluorescent tube) to provide 650 ± 45 lux light intensity.

After 31 days of culture in the induction medium, a half of gram callus was sub-cultured in the dolomite treatment medium. Culture incubation was performed at the exact condition of callus induction culture. The callus in dolomite treatment was harvested after 21 days. The wet and dry weight of callus was determined. The morphology of callus was observed descriptively.

### Anatomical characterization of callus

This method was used to determine the anatomical structure of *Sonchus arvensis* L. callus. The callus at 21 days was used. The technique was the paraffin cloaking method (paraffin embedding). The samples were fixed in standard fixative for paraffin embedding (70% alcohol). Fixed materials were dehydrated serially using alcohol and water, which replaces the water in the material. A paraffin wax of melting point 58–60°C was used for infiltration and embedding. Uniformly thin sections of 10 μm thickness were cut using Leica RM 2145 rotary microtome. Safranin stain was used to localize polysaccharides of the cell wall.

### Leaves and callus extraction

One gram of dried callus and leaves was ground and macerated with 10 mL of ethanol and methanol thrice for 24 h in a closed falcon tube and shaken every 8 h. The liquid was filtered by Whatman no. 1 filter paper and evaporated until the extract’s dried weight became consistent. The extract was used for metabolite profiling by chromatography-mass spectrometry (GC/MS), thin-layer chromatography (TLC) analysis, and in vitro antiplasmodial activity assay. The extract was stored in a refrigerator (±4°C) for further analysis.

### Phytochemical profiling by GC/MS analysis

The phytochemical profiles of extract of callus/leaves were determined by GC/MS analysis. Fifty milligrams of extract were dissolved in 300 μL of methanol and then filtrated with 45 μm filter. The sample’s GC/MS analysis was performed using an Agilent HP 6890 GC Method, Detector Agilent 19091S-433, and HP-5MS 5% Phenyl Methyl Siloxane capillary column (30 m × 0.25 mm, with 0.25 μm film thickness), with 1 mL/min mobile phase. The average velocity was 37 cm/min. The oven temperature was set at 80°C to 300°C at 15°C/min for 2–14 min. Helium was used as the carrier gas at a total flow of 54.2 mL/min. The injector temperature was 300°C, with 1 μL injection volume, 10 μL syringe, and whole running time of 24 min. The interface and mass spectra ion source were maintained at 230°C and 150°C, respectively. The mass spectra were taken at 1624 V, with a mass scan range of 30–550 amu. The identification of compounds was based on mass spectra comparison with Wiley version 275.L and NIST version 02.L library. The quality of compounds above 85% was shown in this study. Each component’s relative percentage was calculated with the relative percentage of the chromatogram’s total peak area.

### Phytochemical screening by TLC analysis

Five milligrams of samples were dissolved in 100 μl of methanol or ethanol. Two microliters of samples were spotted and allowed to dry on TLC plate (Silica gel GF254). The TLC plate was developed using n-hexane: ethyl acetate (4:1) and dried. It was sprayed with ρ-anisaldehyde sulfuric acid and heated. Terpenoids were seen on the TLC plate as a purple-blue node.

### In vitro antiplasmodial activity assay

The antiplasmodial test referred to the method of Rieckmann et al. [22], which was later modified by Wahyuni et al. [11]. The callus extract was tested against *P*. *falciparum* strain 3D7 at ring stage. *P*. *falciparum* cultures were synchronized into the ring stage using 5% sorbitol. The test material was in the form of crude extract from *S*. *arvensis* L. leaves/callus. As much as 10 mg of sample were dissolved in 100 μL of DMSO, dilution was done to obtain 100, 10, 1, 0.1, and 0.01 μg/mL extract concentrations. After 48 hours of incubation, the number of infected erythrocytes was counted. The percentage of parasitemia and inhibition was calculated with the formulas below.

The formula to calculate the percentage of parasitemia:

$$\%\;\mathrm{Parasitemia}=\frac{\sum \mathrm{infected}\;\mathrm{eritrocyte}}{5000\;\mathrm{of}\;\mathrm{total}\;\mathrm{eritrocyte}}\times 100\%$$

The formula to calculate percent inhibition:

$$\%\;\mathrm{Inhibition}=100\%‐[\frac{\mathrm{Xp}}{\mathrm{Xk}}\times 100\%]$$

The formula to calculate percent growth:

% Growth = % parasitemia Un–% parasitemia Do where,

Xp = Treatment parasitemia

Xk = Negative control parasitemia

Un = % parasitemia in each concentration

Do = % parasitemia at the start

## Data analysis

The morpho-anatomical characteristics were analyzed descriptively, while the quantitative data were callus biomass analyzed statistically by ANOVA, followed by Duncan’s test using SPSS 21. Percentage of parasitemia and percentage of inhibition of 3D7 *Plasmodium falciparum* strain were used to analyze the IC_50_ by Probit Analysis.

## Results and discussion

### Callus induction of *Sonchus arvensis* L.

**Fig 1:**
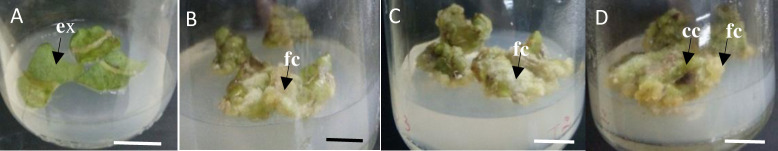
Morphology of *Sonchus arvensis* L. callus on media with 1 mg/L 2,4-D + 0.5 mg/L BAP. A, 10 days. B, 17 days. C, 24 days. D, 31 days. ex. explant. fc. friable callus. cc. compact callus. Bar = 1 cm.

Callus induction is the preparation stage of explants for dolomite treatment. The best callus criteria include fresh callus color, rapid callus formation, and high callus number. The response of callus growth was different for each treatment. The efficiency of *Sonchus arvensis* L. induction was 100% under 2,4-D and BAP treatment. The combination of 1 mg/L 2,4-D and 0.5 mg/L BAP was the best treatment (Fig 1), with the shortest induction time (10 days) and the highest callus number. The callus’ color is yellow, and the callus’ texture is friable (Table 1). This combination was used to produce callus as explant for the next step.

**Table 1:** Efficiency and formation time of *Sonchus arvensis* L. callus induction.

Media treatment (mg/L)	Time of induction (days)	Efficiency induction (%)	Degree of calli	Color of callus	Texture of callus
**MSO**	0	0	–	–	–
**D**_**0**_ **B0**_**,5**_	0	0	–	–	–
**D**_**0**_ **B**_**1**_	14	100	++	Yellowish- white	Friable
**D**_**0,5**_ **B**_**0**_	14	100	++	Brownish-yellow	Friable
**D**_**0,5**_ **B**_**0,5**_	14	100	++	Yellow	Friable
**D**_**0,5**_ **B**_**1**_	10	100	+++	Yellow	Friable
**D**_**1**_ **B**_**0**_	10	100	++++	Yellow	Friable
**D**_**1**_ **B**_**0,5**_	10	100	+++++	Yellow	Friable
**D**_**1**_ **B**_**1**_	10	100	++++	Yellow	Friable

Note: MS0 = without growth regulator, D = 2,4-D in mg/L, and B = BAP in mg/L. -, no callus formed; +, 25% callus of explants; +++, 50% callus of explants; ++++, 75% callus of explants; +++++, 100% callus of explant. Explant = leaf of *Sonchus arvensis* L.

### Morphological structure of *Sonchus arvensis* L. callus on dolomite media

**Fig 2:**
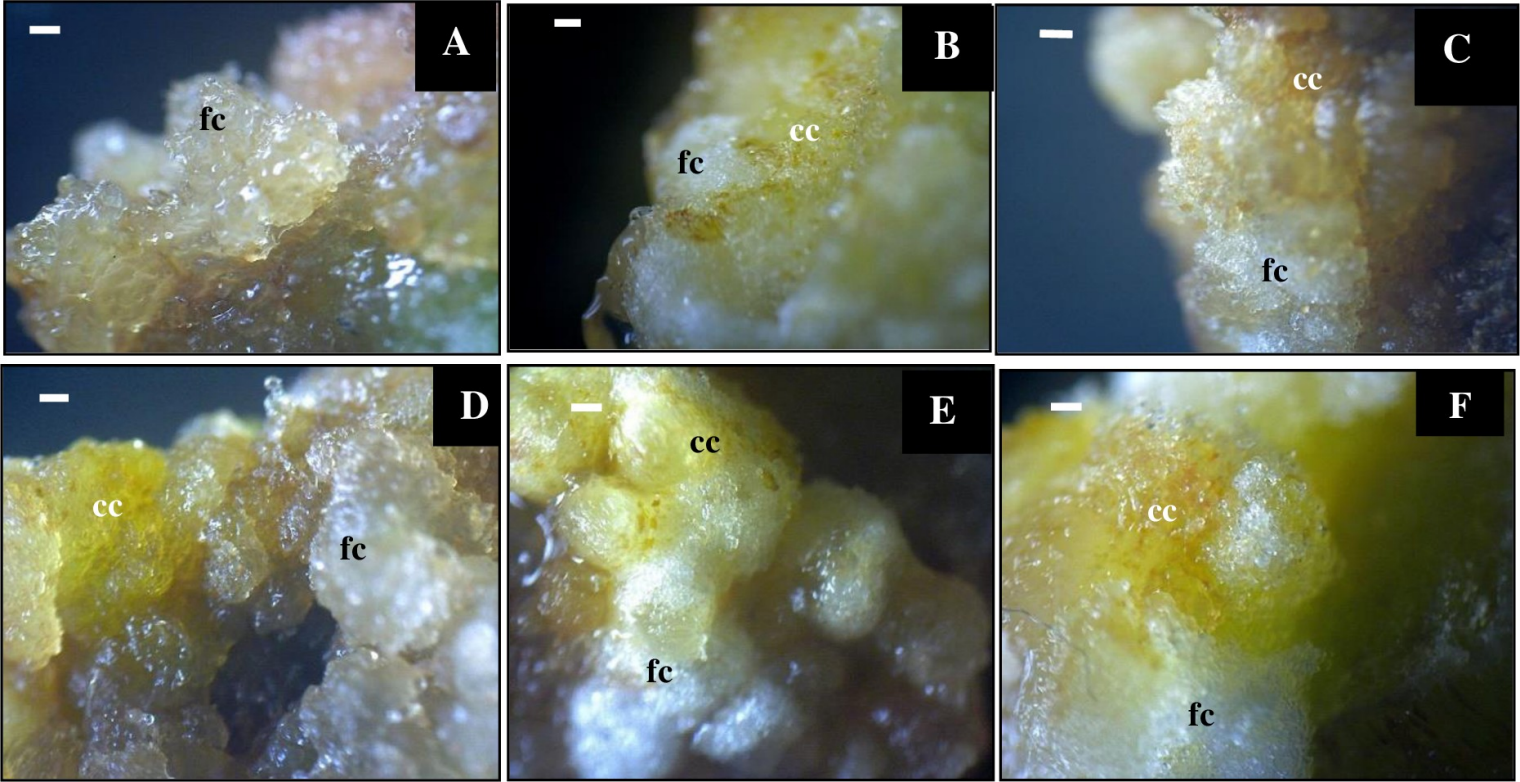
Morphology of *Sonchus arvensis* L. callus on dolomite media. A, control (1 mg/L 2,4-D + 0.5 mg/L BAP). B, 50 mg/L dolomite. C, 75 mg/L dolomite. D, 100 mg/L dolomite. E, 150 mg/L dolomite. F, 200 mg/L dolomite. fc. friable callus. cc. compact callus. Bar = 100 μm.

The *Sonchus arvensis* L. callus from 1 mg/L 2,4-D + 0.5 mg/L BAP medium was used as explants in dolomite media with concentrations of 0, 50, 75, 100, 150, and 200 mg/L. The callus was observed for three weeks (Table 2). The callus in different dolomite media showed different morphological characters. The texture of callus produced after subculture in dolomite media changed into compact compared to the control (1 mg/L 2.4-D + 0.5 mg/L BAP). However, it still had a friable part of newly formed callus (Fig 2).

**Table 2:** Degree, texture, and color of *Sonchus arvensis* L. callus on dolomite media.

Weeks	Media (mg/L)	Degree of callus	Morphology	
			Texture	Color
**0**	2,4-D1 + BAP0,5	++	Friable	Yellow
	D50	++	Friable	Yellow
	D75	++	Friable	Yellow
	D100	++	Friable	Yellow
	D150	++	Friable	Yellow
	D 200	++	Friable	Yellow
**1**	2,4-D1 + BAP0,5	+++	Friable	Brownish-yellow
	D50	+++	Friable, compact	Yellow and some brownish-yellow
	D75	++++	Friable, compact	Bright yellow to brownish-yellow
	D100	+++++	Friable, compact	Bright yellow to brownish-yellow
	D150	++++++	Friable, compact	White and bright yellow to brownish
	D 200	+++++++	Friable, compact	White and bright yellow to brownish
**2**	2,4-D1 + BAP0,5	++++	Friable	Brownish-yellow
	D50	++++	Friable, compact	Brownish-yellow
	D75	+++++	Friable, compact	Reddish-yellow and white
	D100	++++++	Friable, compact	White and bright yellow to brownish
	D150	+++++++	Friable, compact	White and bright yellow to brownish
	D 200	++++++++	Friable, compact	White and bright yellow to brownish
**3**	2,4-D1 + BAP0,5	++++	Friable	Brownish-yellow
	D50	+++++	Friable, compact	White and brownish-yellow
	D75	++++++	Friable, compact	White and reddish-yellow
	D100	+++++++	Friable, compact	White and bright yellow to brownish
	D150	+++++++++	Friable, compact	White and bright yellow to brownish
	D 200	++++++++	Friable, compact	White and bright yellow to brownish

Note: 2,4-D = 2,4-dichlorophenoxyacetic acid, BAP = 6-benzyl amino purine and D = Dolomite; +: the degree of callus growth, + +, 25% callus of explants; ++++, 50% callus of explants; ++++++, 75% callus of explants; ++++++++, 100% callus of explant. Explant = callus of *Sonchus arvensis* L. under 1 mg/L 2,4-D and 0.5 mg/L BAP treatment.

Friable calluses, which were first observed in all treatments yellowish-white and greenish-white transparent (Figs 1 and 2), were formed through cells’ growth at a small site with loose cell interactions. Previous studies have reported that 2,4-D stimulated cell elongation by increasing the cell wall’s plasticity to become loose, allowing water to flow into the inner cell by osmosis, causing the cell to become elongated quickly [23, 24]. Thus, friable calluses contain a lot of water because the cell wall is not yet lignified, and the cells can be easily separated from each other. A friable callus from an explant has loose cell interactions and can be easily detached using tweezers [25].

Compact calluses were produced in this study after three weeks of culture. Compact calluses are composed of tight cells that are difficult to separate, relatively whitish-yellow to brown, with a smooth surface. Our finding agreed with other callus induction reports for secondary metabolite production [23].

The callus on the control media (without dolomite) was brownish-yellow, while the callus on the dolomite media was transparent, white, bright yellow, and reddish to brownish (Fig 2 and Table 2). The color change of calluses indicates cell activity during cell division [26]. In addition, the color of calluses becomes brown because they produce phenolic compounds that can be toxic to plants and stop growth [27]. Further discoloration of the callus, from white to dark brown, indicates the low cleavage activity of callus cells [28]. Taranto et al. [29] found that callus browning is caused by polyphenolic compounds present when the explants were wounded. The enzymatic browning reaction of phenolic compounds was oxidized by polyphenol oxidase, peroxidase, or air exposure.

**Fig 3:**
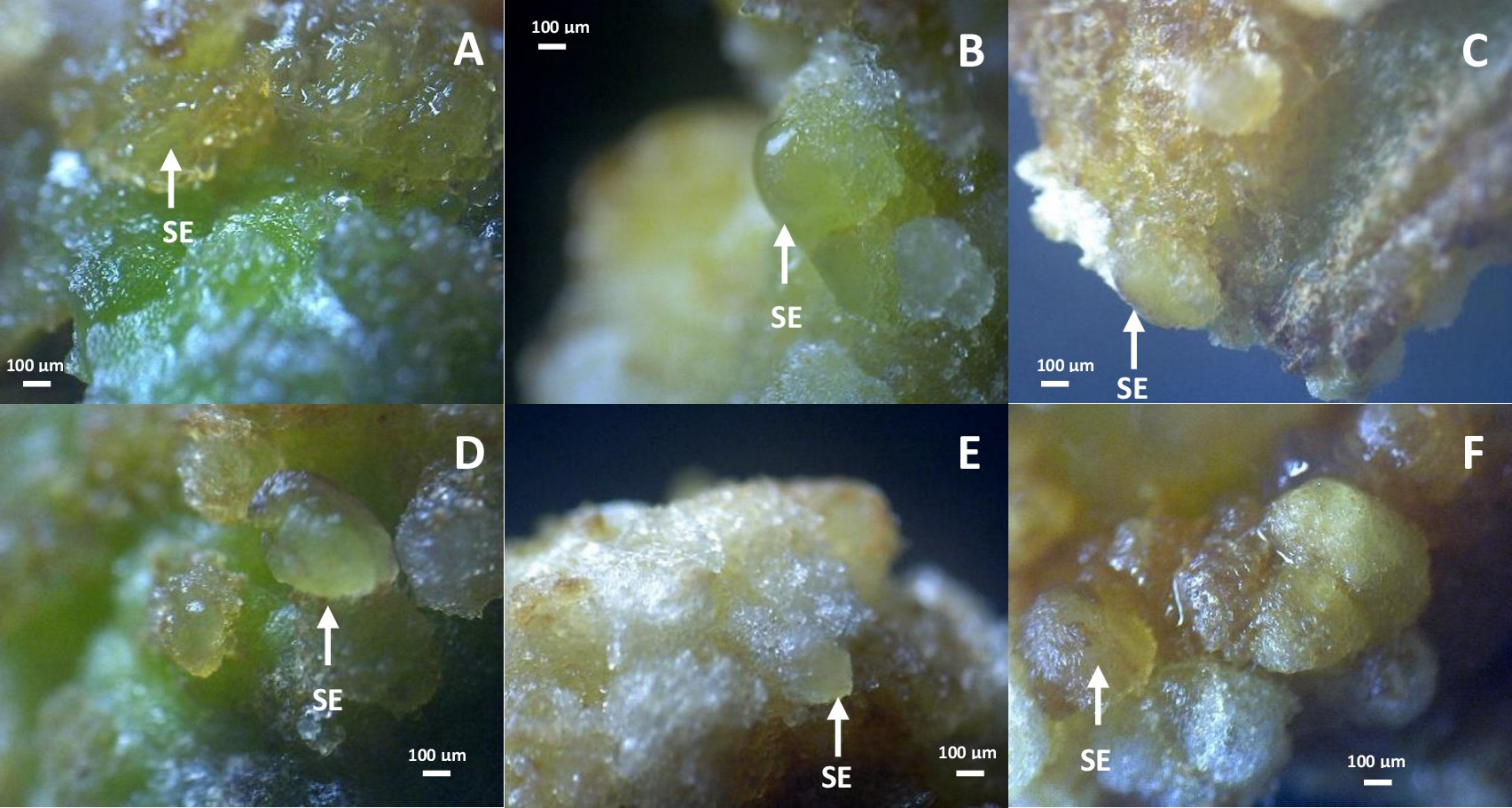
Embryogenic callus of *Sonchus arvensis* L. on dolomite media. A, control (1 mg/L 2,4-D + 0.5 mg/L BAP). B, 50 mg/L dolomite. C, 75 mg/L dolomite. D, 100 mg/L dolomite. E, 150 mg/L dolomite. F, 200 mg/L dolomite. SE: somatic embryo.

Embryonic callus was found in all dolomite media in different amounts. The higher the dolomite concentration, the more embryonic callus is produced. The embryonic callus is shown with white arrows (Fig 3). The embryonic callus is characterized by a change in the callus color to transparent or yellowish-white, prominent cell formation, and more compact texture. Adding dolomite triggers embryonic callus because it contains Ca2+ and Mg2+ [30], supporting plants’ growth and development [31].

### Anatomy of *Sonchus arvensis* L. callus under dolomite treatment

**Fig 4:**
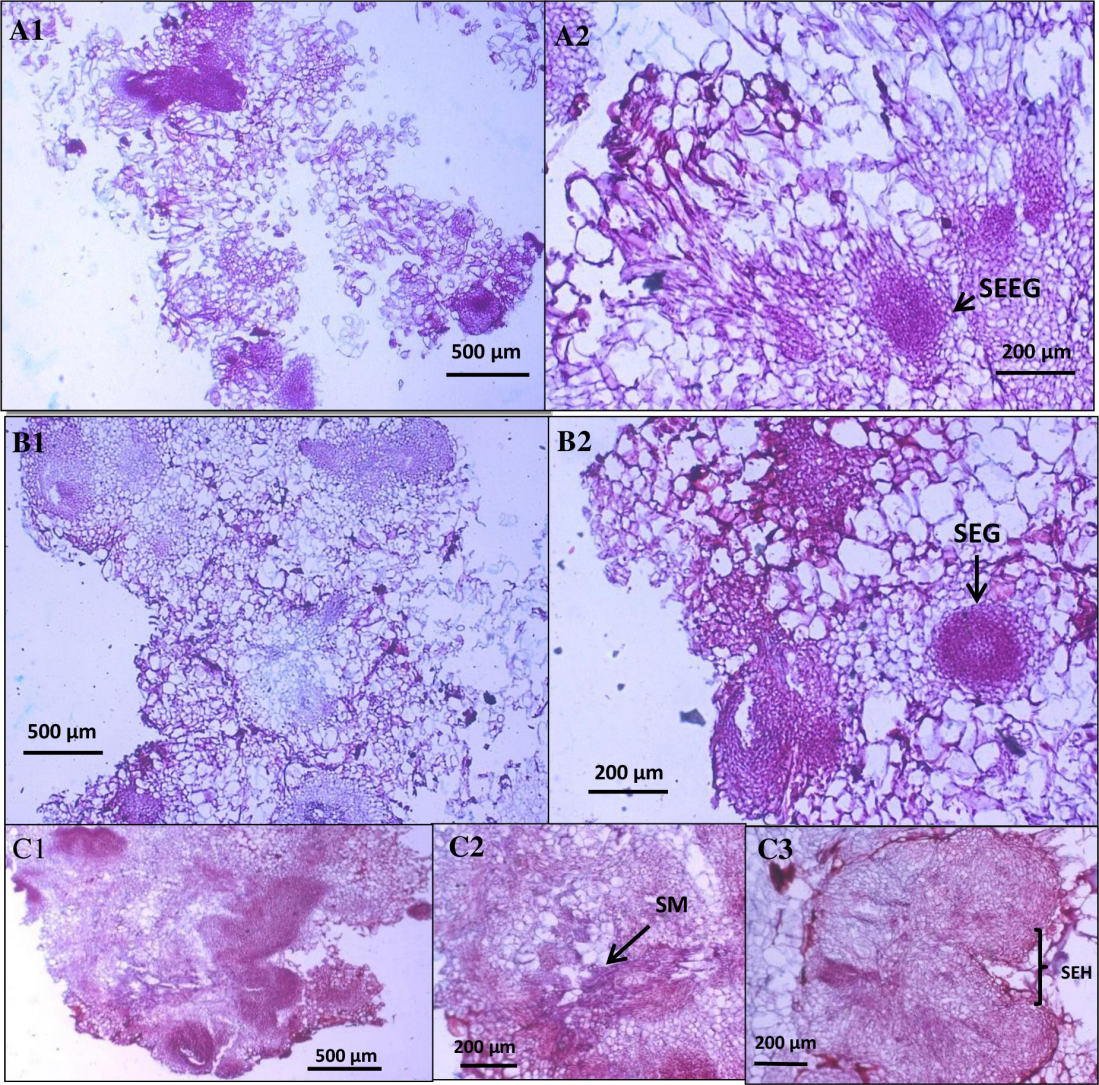
Cross-section of *Sonchus arvensis* L. callus on dolomite media. A1–A2, control callus from 1 mg/L 2,4-D + 0.5 mg/L BAP (A1: filled with parenchymatous cells, A2: containing somatic embryo in early globular stages/SEEG). B1–B2, callus from 50 mg/L dolomite (B1: filled with parenchymatous cells, B2: containing somatic embryo in globular stages/SEG). C1–C3, callus from 75 mg/L dolomite (C1: filled with parenchymatous cells, C2: secondary metabolites (SM) accumulation in parenchymatous cells, and C3: somatic embryo in hearth stages/SEH).

**Fig 5:**
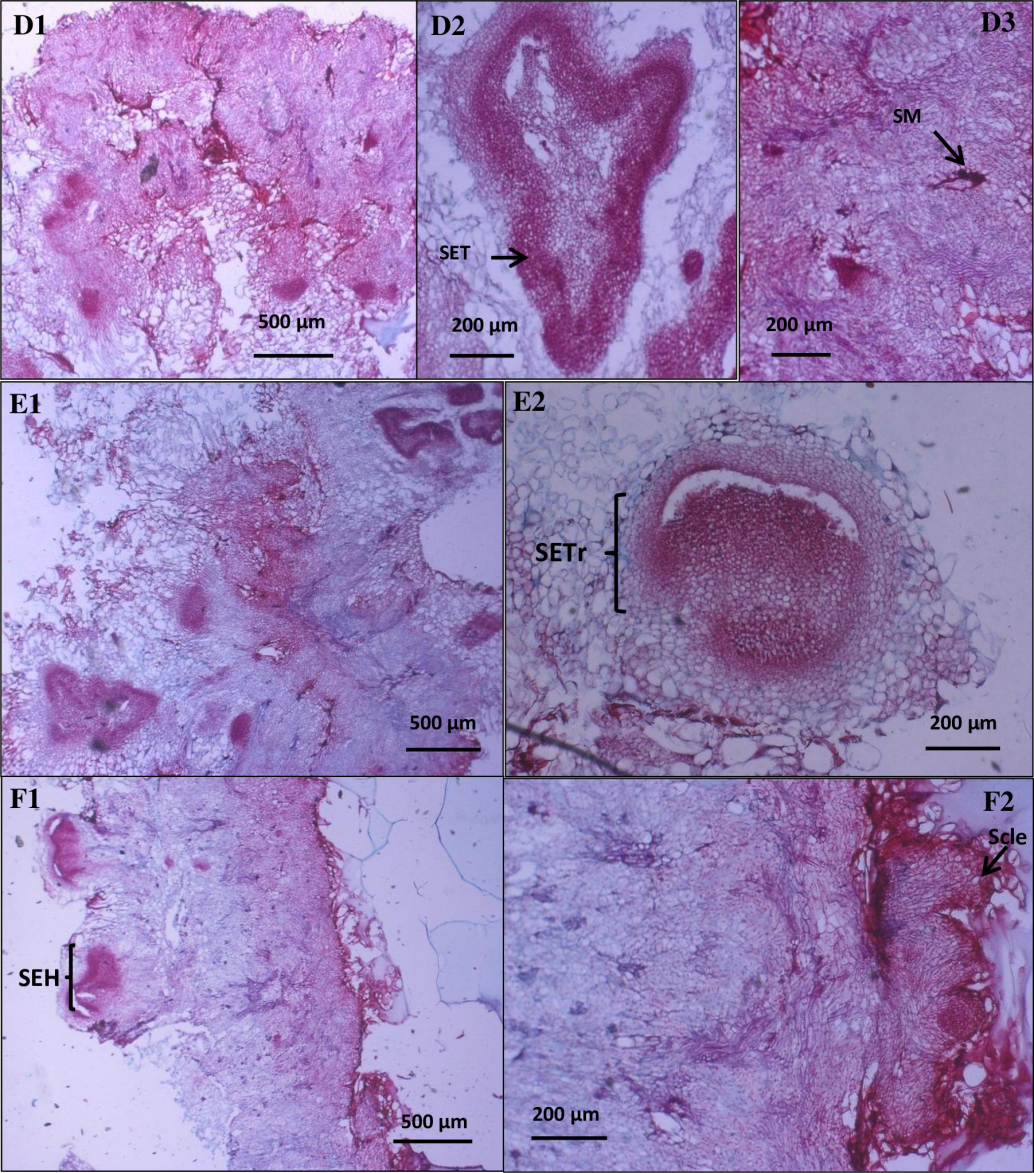
Cross-section of *Sonchus arvensis* L. callus under dolomite treatment. D1–D3, callus from 100 mg/L dolomite (D1: filled with small parenchymatous cells with thick cell wall and sclerenchymatous cells, D2: containing somatic embryo torpedo stages/SET, and D3: secondary metabolite (SM accumulation in interstitial cell). E1–E2, callus from 150 mg/L dolomite (E1: filled with small parenchyma cells with thick cell wall, E2: containing somatic embryo in transition stages (SETr) from globular to heart stages). F1–F2, callus from 200 mg/L dolomite (F1: filled with small parenchymatous cells with thick cell wall, F2: there were sclerenchymatous cells on the edge of callus).

In the first stage, the leaf explant from *Sonchus arvensis* L. was inoculated on MS medium with various levels of growth regulator hormones 2,4-D and BAP. The best callus formation and growth rate have come from medium MS supplemented with 1 mg/L 2,4-D and 0.5 mg/L BAP, used as a control in dolomite treatment. The *S*. *arvensis* L. callus used in this research was cultivated for 31 days. It was then sub-cultured in the new medium (MS medium with various dolomite concentrations) and incubated for three weeks. Callus growth can be seen in Figs 4 and 5.

The anatomy of callus in the control medium was friable, mainly composed of large parenchymatous cells containing many somatic embryos in early globular stage (Fig 4A1 and 4A2). According to Azeez et al. [32], friable callus tissue was parenchymatous and meristematic. The cells of callus from control were distributed uniformly in a pro-embryogenic mass, similar to Ramires and Silva [33].

The callus from 50 mg/L dolomite medium was solid. It is composed of small parenchymatous cells, meristematic cells (Fig 4B1), and globular embryos (Fig 4B2). The embryonic callus consisted of a semi-transparent mass of greenish cells [34].

The callus from 75 mg/L dolomite medium was solid and mostly composed of small parenchymatous and meristematic cells (Fig 4C1). The globular and hearth embryos also developed from the callus (Fig 4C3). The secondary metabolite was found in these calluses and accumulated in the parenchymatous cells (Fig 4C2).

The callus from 100 mg/L, 150 mg/L, and 200 mg/L dolomite medium is also mostly composed of meristematic and sclerenchymatous cells. These calluses also have a somatic embryo, starting from the globular phase, heart, and torpedo phases. Secondary metabolites were also found in this callus, especially in the parenchymatous cells (Fig 5). This fact confirmed the possibility of generating a plant from callus under dolomite treatment. It seems like Bacinni and Tani [35] study on *Nicotiana tabacum* L. callus.

The callus grown on dolomite medium has different characters that can be seen in Table 3. The higher the dolomite concentration, the smaller the cell size and more somatic embryos and secondary metabolites. Based on our result, the first cell formed in the early stage of callus formation was the parenchymatous cell. It is similar to the study by Daksyayini et al. [34] and Ariati et al. [12] that observed *Chicorium intybus* callus. Compact callus has small cell size, with dense cytoplasm, large nucleus, and starch granules.

**Table 3:** Comparison of the anatomical characteristics of *Sonchus arvensis* L. callus on dolomite media.

Character	Control	Dolomite (mg/L)				
		50	75	100	150	200
**Callus type**	Friable	Compact	Compact	Compact	Compact	Compact
**Cell structure**	Parenchyma (+++), meristem (+)	Parenchyma (++), meristem (++)	Parenchyma (+), meristem (++)	Parenchyma (+), meristem (+++)	Parenchyma (+), meristem (+++), schelenchyma (+)	Parenchyma (+), meristem (+++), schelenchyma (++)
**Thickening of Cell wall**	−	+	++	+++	+++	++++
**Cell density**	Not solid	Solid (+)	Solid (++)	Solid (++)	Solid (+++)	Solid (++++)
**Somatic embryo**	Globular stage (+)	Globular stage (++)	Globular stage (+) and heart stage (+)	Globular stage (+) and heart stage (++), and torpedo stage (+)	Globular stage (+), heart stage (+), and torpedo stage (+)	Globular stage (+), heart stage (++), and torpedo stage (++)
**Secondary metabolite**	−	−	+	++	+++	++++

Note: Control: callus under 2,4-D (1 mg/L) + BAP (0.5 mg/L) treatment; -: no characters appearance; +: degree of characters appearances.

### Biomass of *Sonchus arvensis* L. callus on dolomite media

Three-week-old calli grown on dolomite media were harvested, and their wet and dry weight were determined to examine the level of cell proliferation and cell biomass. Callus growth under dolomite treatment was very diverse. The 150 mg/L dolomite treatment produced the highest fresh weight of 0.590 ± 0.136 g and dry weight of 0.074 ± 0.008 g, followed by 200 mg/L dolomite treatment with 0.372 ± 0.120 g fresh weight and 0.052 ± 0.018 g dry weight; 100 mg/L = 0.368 ± 0.037 g (fresh weight) and 0.046 ± 0.015 g (dry weight); 75 mg/L = 0.344 ± 0.129 g (fresh weight) and 0.038 ± 0.008 g (dry weight); 50 mg/L = 0.327 ± 0.018 g (fresh weight) and 0.036 ± 0.003 g (dry weight); and control (0 mg/L) = 0.297 ± 0.094 g (fresh weight) and 0.032 ± 0.013 g (dry weight). Based on the Duncan’s Multiple Range Test (DMRT) statistical test results at 5% significance level, the average wet and dry weight in 150 mg/L dolomite treatment was significantly different from all treatments (S1 Table and Table 4).

**Table 4:** The fresh and dry weight of *Sonchus arvensis* L. callus on dolomite media after 21 days cultured.

Dolomite (mg/L)	Fresh Weight (g)	Dry weight (g)
**0**	0.297 ± 0.094^a^	0.032 ± 0.013 ^a^
**50**	0.327 ± 0.018 ^a^	0.036 ± 0.003 ^a^
**75**	0.344 ± 0.129 ^a^	0.038 ± 0.008 ^a^
**100**	0.368 ± 0.037 ^a^	0.046 ± 0.015 ^a^
**150**	0.590 ± 0.136 ^b^	0.074 ± 0.008 ^b^
**200**	0.372 ± 0.120 ^a^	0.052 ± 0.018 ^a^

Note: The values followed by the same letter show no significant difference (α = 5%) in the Duncan’s Multiple Range Test (DMRT).

This study is the first to apply dolomite to tissue culture. Callus growth increased with increasing dolomite concentration and decreased to 200 mg/L. This result is in accordance with the study of Chutichudet et al. [20] on grand rapids lettuce. Hence, dolomite is widely applied in agricultural systems because of its content [36]. In general, dolomite has two significant effects: increased environmental pH from the decrease in H^+^ ions caused by dolomite hydrolysis and Ca^2+^ and Mg^2+^ ions source. Calcium and magnesium play essential roles in plant growth and development. Ca2+ is required for structural parts of cell walls and membranes as a divalent cation, while Mg^2+^ is a structural constituent of chlorophyll [31].

### Metabolite profiles of *Sonchus arvensis* L. callus extract on dolomite media

**Fig 6:**
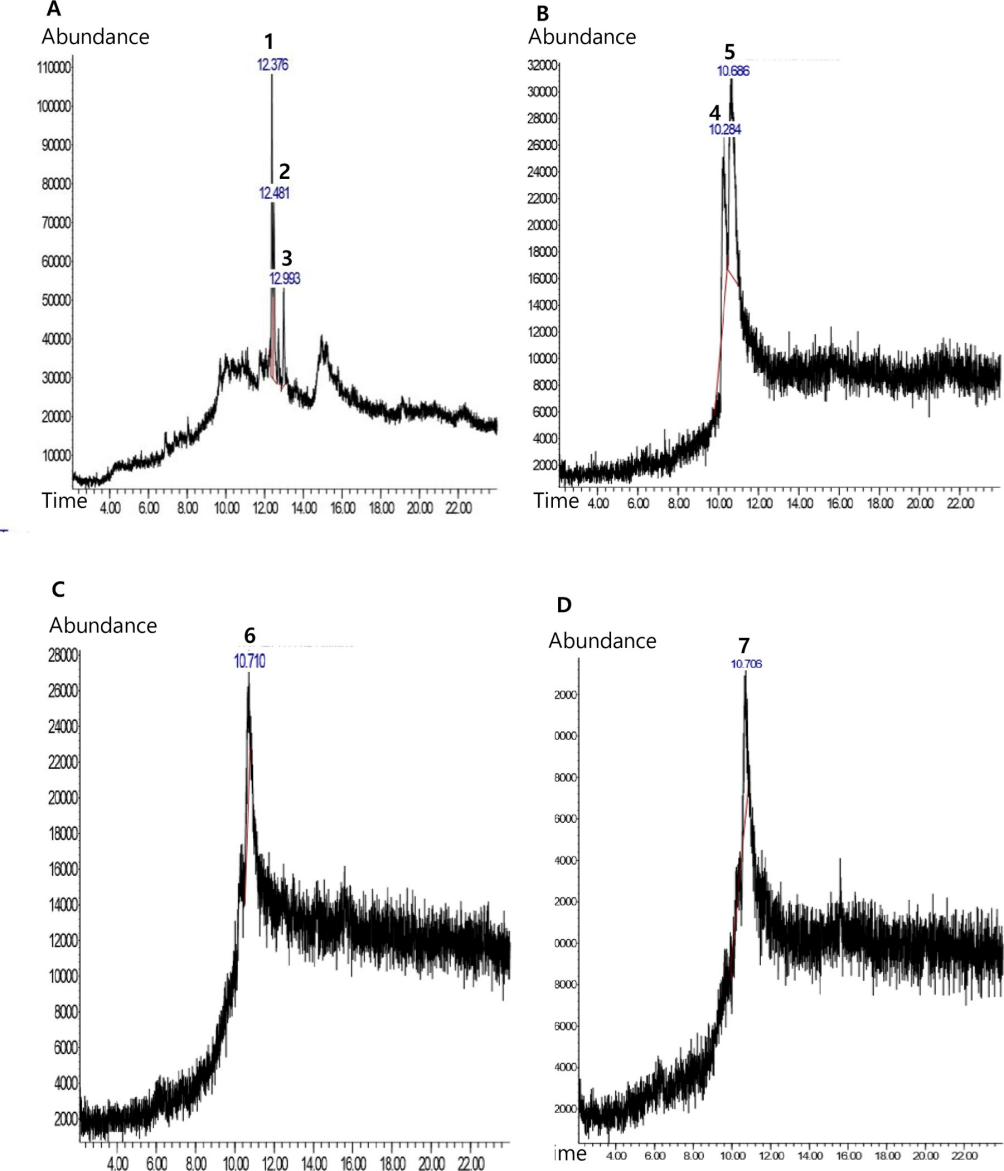
Chromatogram of ethanolic extracts of *Sonchus arvensis* L. callus in different dolomite concentrations. A, leaves. B, control (1 mg/L 2,4-D + 0.5 mg/L BAP). C, 75 mg/L dolomite. D, 150 mg/L dolomite. 1. Phytol, 2. 2-Pentadecanone, 3. 1,2-dimethyl-3-(1-methyl ethyl) cyclopentane, 4. Methyl beta-D-glucopyranoside, 5. Pelargonic acid, 6. Decanoic acid, 7. Hexadecanoic acid.

Chemical analysis by GC-MS was used to determine the metabolite profile of callus extract. *Sonchus arvensis* L. ethanolic extract of leaves from a wild plant, callus on without dolomite medium(control), and callus on dolomite medium (75 mg/L dolomite, and 150 mg/L dolomite treatments) were analyzed (Table 5 and Fig 6). The 75 mg/L and 150 mg/L dolomite treatments were selected based on callus morpho-anatomy and weight.

**Table 5:** Metabolite profiles of *Sonchus arvensis* L. callus ethanolic extract.

Sample/extract	Compound name	Retention time (min)	Area %	Compound group	Bioactivities [40]
Leaves	Phytol	12,375	47,75	Prenol lipids, acyclic diterpenoids	Antibacterial, antimicrobe, antifungal
	2-Pentadecanone	12,483	35,33	Organooxygen compounds, ketones	Antioxidant, antibacterial
	1,2-dimethyl-3-(1-methylethyl)cyclopentane	12,992	16,91	Prenol lipids, monocyclic monoterpenoids	Antibacterial
Control callu**s** (2,4-D 1 mg/L + BAP 0,5 mg/L)	Methyl beta-D-glucopyranoside	10,283	29,31	Organooxygen compounds,O-glycosyl compounds	Additive
	Pelargonic acid	10,683	70,69	Fatty acid, medium-chain fatty acid	Antifungal, herbicide, insecticide, growth regulator substance, acaricide
Dolomite 75 mg/L callus	Decanoic acid	10,712	100	Fatty acid, medium-chain fatty acid	Antifungal, Antifungi, herbicide, insecticide, growth regulator substance, acaricide herbicide, insecticide, growth regulator substance, acaricide
Dolomite 150 mg/L callus	Hexadecanoic acid	10,706	100	Fatty acid, long-chain fatty acid	Antibacterial, antifungal, antioxidant, pesticides

Note: The content of the table in detail is supported the chromatogram available in (S2–S5 Tables).

The compounds detected were different in each sample. *Sonchus arvensis* L. leaves contain phytol, 2-pentadecanone, and 1,2-dimethyl-3-(1-methyl ethyl) cyclopentane compounds. Phytol compound had the highest area of 47.75%, with a retention time of 12.375 (S2 Table). The compounds detected in the ethanol extract of the control callus (1 mg/L 2,4-D + 0.5 mg/L BAP) were methyl beta-D-glucopyranoside and pelargonic acid, which had the largest area of 70.69%, with a retention time of 10.683 (S3 Table).

The compound that was detected in the ethanol extract of 75 mg/L dolomite treatment was decanoic acid, which was found at a retention time of 10,712, with an area of 100% (S4 Table). Hexadecanoic acid was found in 150 mg/L dolomite callus ethanol extract at a retention time of 10,078, with an area of 100% (S5 Table).

GC-MS chromatogram of ethanol extract revealed three compounds in leaf, two compounds in control callus (without dolomite media), and one compound in callus on dolomite media. The compounds detected using this method in each sample were different. The secondary metabolite profiles of calli were influenced by nutrient, temperature, light, growth-regulating substance, and carbon source [26, 37, 38]. This result is different from the study results of Syamkumar et al. [39], which states that the metabolite profiles of callus compounds and leaves are identical.

### In vitro antiplasmodial activity of *Sonchus arvensis* L. callus extract on dolomite media

The parasite used in this test was *Plasmodium falciparum* strain 3D7. The percentage of parasitemia, growth and parasite inhibition by callus extract was calculated, and probit analysis was performed to determine the minimum inhibitory concentration (IC_50_) (S6 Table). Based on probit analysis, it was found that the IC_50_ of methanolic and ethanolic extracts of leaves, callus from control (1 mg/L 2,4-D + 0.5 mg/L BAP) and callus from 150 mg/L dolomite treatment are 17.78 μg/mL, 27.09 μg/mL, 12.469 μg/mL, >10 μg/mL, 5.037 μg/mL and 5.944 μg/mL, respectively (Table 6). Extracts with IC_50_ of 1–10 μg/mL are effective ingredients for antimalarial drugs [41, 42]. In a study in China, extracts with an IC_50_ of 0.008–15.38 μg/mL were used as a malaria drug ingredient [43].

**Table 6:** Percentage of inhibition and the IC_50_ of antiplasmodial activity of *Sonchus arvensis* L. callus against *Plasmodium falciparum* strain 3D7.

Extract	Negative control (μg/ml)	Average inhibition percentage (%)					IC_50_ (μg/ml)
		100 (μg/ml)	10 (μg/ml)	1 (μg/ml)	0,1 (μg/ml)	0,01 (μg/ml)	
**Leave methanolic extract**	−	100	44.30	27.69	7.17	5.54	17.78
**Leave ethanolic extract**	−	100	36.69	31.65	6.83	0	27.09
**2,4-D 1 mg/L + BAP 0,5 mg/L callus methanolic extract**	−	83.92	35.69	17.25	9.02	0	12.469
**2,4-D 1 mg/L + BAP 0,5 mg/L callus ethanolic extract**	−	100	9.69	0	0	0	>10
**Dolomite 150 mg/L callus methanolic extract**	−	96.56	53.95	17.87	9.28	0	5.037
**Dolomit 150 mg/L callus ethanolic extract**	−	100	60.07	21.58	11.51	0	5.944

### Thin-layer chromatography of *Sonchus arvensis L*. callus

**Fig 7:**
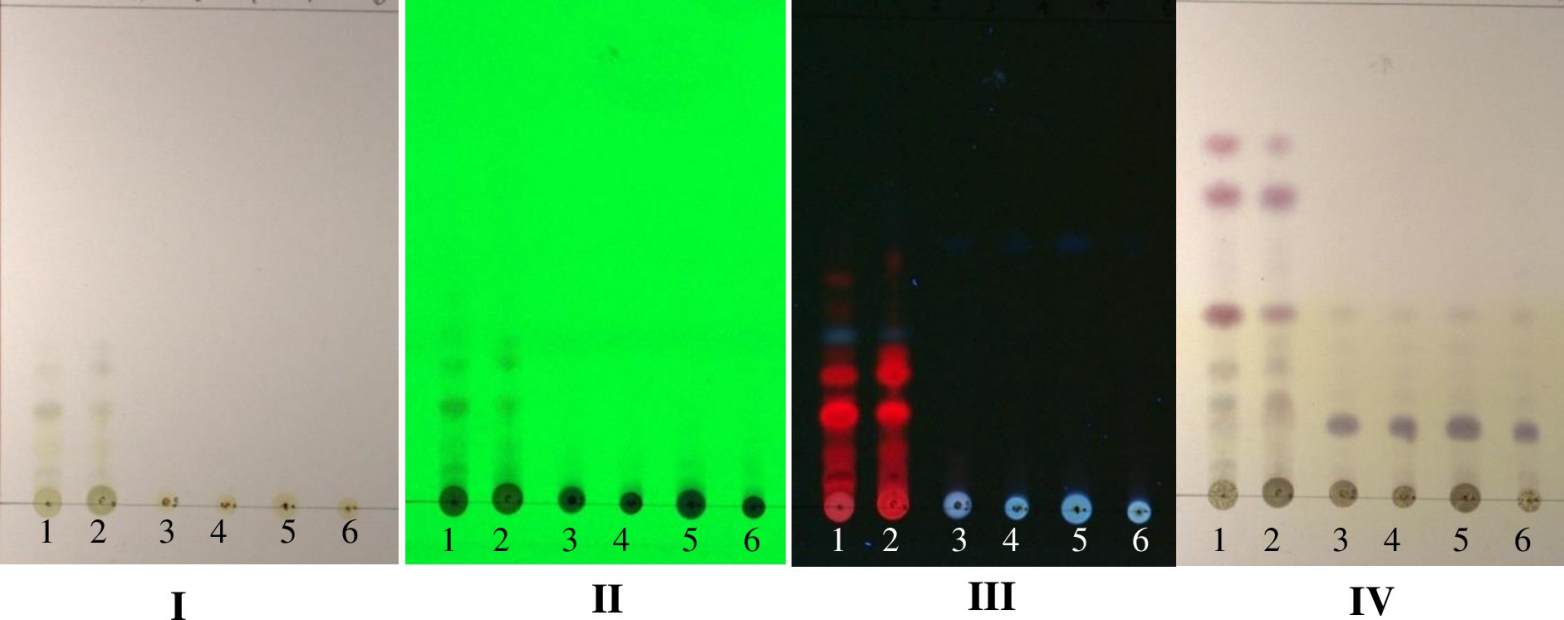
TLC chromatogram of *Sonchus arvensis* L. callus extract. I, after running with solvent (4 n-hexane: ethyl acetate). II, UV 254 nm. III, UV 366 nm. IV, after being sprayed with anisaldehyde sulfuric acid (the purple spot is terpenoid). 1, leaf methanolic extract. 2, leaf ethanolic extract. 3, control (1 mg/L 2,4-D + 0.5 mg/L BAP ethanolic extract). 4, 75 mg/L dolomite ethanolic extract. 5, 150 mg/L dolomite ethanolic extract. 6, 200 mg/L dolomite ethanolic extract.

Callus extracts from various treatments underwent TLC analysis. One visible spot on daylight and under 254-nm UV light with retention/retardation factor (Rf) value = 0.17 for ethanolic and methanolic extracts of leaves (wild plant), but it was not seen in control (without dolomite media) and treated callus extracts (dolomite media). Under 366-nm UV light on ethanolic and methanolic leaves extract, there were seven separate stains with Rf values of 0.07, 0.10, 0.19, 0.26, 0.35, 0.42, and 0.47. Simultaneously, there was one stain on the control, and the dolomite treatment callus extract was purple with Rf value of 0.54. After staining using ρ-anisaldehyde sulfuric acid, five separate purple stains were seen on leaf extract, with Rf values of 0.17, 0.25, 0.37, 0.62, and 0.71. Simultaneously, the callus extract from the control and dolomite treatment showed one purple stain with Rf value of 0.15 (Fig 7). TLC was used to separate plant extracts’ secondary metabolites [44]. The Rf value indicates a significant diversity of terpenoid compounds separated from various extracts [45].

## Conclusions

This study showed the effects of dolomite on growth, metabolite production, morpho-anatomy, and *Sonchus arvensis* L. callus’ antiplasmodial activity. Our results significantly suggested that 150 mg/L dolomite could enhance the growth and antiplasmodial activity of *S*. *arvensis* L. callus. The metabolite profiles of the callus extract are also different from the control and wild plant extract. This finding is a starting point for future studies. For future pharmaceutical purposes, they could produce antiplasmodial material or other phytochemicals by in vitro culture. Additionally, the establishment of callus cultures was recommended because they could be a source of higher bioactive metabolites.

## Supporting information

S1 Table SPSS analysis of biomass (fresh and dry weight) *Sonchus arvensis* L. callus by adding dolomite. (PDF)

S2 Table GC-MS analysis of *Sonchus arvensis* L. leaf. (PDF)

S3 Table GC-MS analysis of *Sonchus arvensis* L. callus under 1 mg/L 2,4-D and 0.5 mg/L BAP treatment. (PDF)

S4 Table GC-MS analysis of *Sonchus arvensis* L. callus under 1 mg/L 2,4-D, 0.5 mg/L BAP, and 75 mg/L dolomite treatment. (PDF)

S5 Table GC-MS analysis of *Sonchus arvensis* L. callus under 1 mg/L 2,4-D, 0.5 mg/L BAP, and 150 mg/L dolomite treatment. (PDF)

S6 Table Probit analysis of in vitro antimalaria of *Sonchus arvensis* L. callus under dolomite treatment. (PDF)

S1 Raw images (PDF)
