# Supplementary material for: Growth, secondary metabolite production, and in vitro antiplasmodial activity of Sonchus arvensis L. callus under dolomite [CaMg(CO3)2] treatment
Source: PLoS One. 2021 Aug 20;16(8):e0254804. doi: 10.1371/journal.pone.0254804 (PMC8378700; doi:10.1371/journal.pone.0254804)
Supplement: S1 Table — (PDF) [file pone.0254804.s001.pdf]

SPSS ANALYSIS OF BIOMASS (FRESH AND DRY WEIGHT) *Sonchus arvensis* L. CALLUS BY ADDING DOLOMITE

FRESH WEIGHT

Normality Test

Tests of Normality

		Kolmogorov-Smirnov[a]			Shapiro-Wilk		
	Dolomite	Statistic	df	Sig.	Statistic	df	Sig.
Fresh_Weight	0 ppm	.272	4	.	.858	4	.252
	50 ppm	.239	4	.	.949	4	.710
	75 ppm	.281	4	.	.878	4	.330
	100 ppm	.302	4	.	.825	4	.156
	150 ppm	.214	4	.	.960	4	.777
	200 ppm	.345	4	.	.765	4	.053

a. Lilliefors Significance Correction

Homogeneity Test

Test of Homogeneity of Variances

		Levene Statistic	df1	df2	Sig.
Fresh_Weight	Based on Mean	3.528	5	18	.021
	Based on Median	1.926	5	18	.140
	Based on Median and with adjusted df	1.926	5	8.023	.195
	Based on trimmed mean	3.279	5	18	.028

ANOVA Test

ANOVA

Fresh_Weight

	Sum of Squares	df	Mean Square	F	Sig.
Between Groups	.221	5	.044	4.369	.009
Within Groups	.182	18	.010		
Total	.403	23			

Post hoc Test

Multiple Comparisons

Dependent Variable: Fresh Weight

LSD

(I) Dolomite	(J) Dolomite	Mean Difference (I-J)	Std. Error	Sig.	95% Confidence Interval Lower Bound	Upper Bound
0 ppm	50 ppm	-.0297000	.0711474	.681	-.179175	.119775
	75 ppm	-.0468000	.0711474	.519	-.196275	.102675
	100 ppm	-.0711250	.0711474	.331	-.220600	.078350
	150 ppm	-.2933250*	.0711474	.001	-.442800	-.143850
	200 ppm	-.0877750	.0711474	.233	-.237250	.061700
50 ppm	0 ppm	.0297000	.0711474	.681	-.119775	.179175
	75 ppm	-.0171000	.0711474	.813	-.166575	.132375
	100 ppm	-.0414250	.0711474	.568	-.190900	.108050
	150 ppm	-.2636250*	.0711474	.002	-.413100	-.114150
	200 ppm	-.0580750	.0711474	.425	-.207550	.091400
75 ppm	0 ppm	.0468000	.0711474	.519	-.102675	.196275
	50 ppm	.0171000	.0711474	.813	-.132375	.166575
	100 ppm	-.0243250	.0711474	.736	-.173800	.125150
	150 ppm	-.2465250*	.0711474	.003	-.396000	-.097050
	200 ppm	-.0409750	.0711474	.572	-.190450	.108500
100 ppm	0 ppm	.0711250	.0711474	.331	-.078350	.220600
	50 ppm	.0414250	.0711474	.568	-.108050	.190900
	75 ppm	.0243250	.0711474	.736	-.125150	.173800
	150 ppm	-.2222000*	.0711474	.006	-.371675	-.072725
	200 ppm	-.0166500	.0711474	.818	-.166125	.132825
150 ppm	0 ppm	.2933250*	.0711474	.001	.143850	.442800
	50 ppm	.2636250*	.0711474	.002	.114150	.413100
	75 ppm	.2465250*	.0711474	.003	.097050	.396000
	100 ppm	.2222000*	.0711474	.006	.072725	.371675
	200 ppm	.2055500*	.0711474	.010	.056075	.355025
200 ppm	0 ppm	.0877750	.0711474	.233	-.061700	.237250
	50 ppm	.0580750	.0711474	.425	-.091400	.207550
	75 ppm	.0409750	.0711474	.572	-.108500	.190450
	100 ppm	.0166500	.0711474	.818	-.132825	.166125
	150 ppm	-.2055500*	.0711474	.010	-.355025	-.056075

*. The mean difference is significant at the 0.05 level.

Duncan Test

Fresh_Weight

	Dolomite	N	Subset 1	Subset 2
Duncan[a]	0 ppm	4	.2969	
	50 ppm	4	.3266	
	75 ppm	4	.3437	
	100 ppm	4	.3680	
	200 ppm	4	.3847	
	150 ppm	4		.5902
	Sig.		.281	1.000

Means for groups in homogeneous subsets are displayed.
Based on observed means.
The error term is Mean Square(Error) = .010.

a. Uses Harmonic Mean Sample Size = 4.000.

DRY WEIGHT

Normality Test

Tests of Normality

	Dolomite	Kolmogorov-Smirnov[a] Statistic	df	Sig.	Shapiro-Wilk Statistic	df	Sig.
Dry_Weight	0 ppm	.278	4	.	.838	4	.189
	50 ppm	.404	4	.	.719	4	.019
	75 ppm	.285	4	.	.809	4	.118
	100 ppm	.284	4	.	.875	4	.317
	150 ppm	.247	4	.	.906	4	.462
	200 ppm	.306	4	.	.833	4	.176

a. Lilliefors Significance Correction

- Berdistribusi Normal : BIla signifikan >0.05

Homogeneity Test

Test of Homogeneity of Variances

		Levene Statistic	df1	df2	Sig.
Dry_Weight	Based on Mean	1.714	5	18	.182
	Based on Median	.893	5	18	.507
	Based on Median and with adjusted df	.893	5	7.835	.529
	Based on trimmed mean	1.496	5	18	.240

ANOVA Test

ANOVA

Dry_Weight

	Sum of Squares	df	Mean Square	F	Sig.
Between Groups	.005	5	.001	6.252	.002
Within Groups	.003	18	.000		
Total	.007	23			

Post Hoc Test

Multiple Comparisons

Dependent Variable: Dry_Weight

LSD

(I) Dolomite	(J) Dolomite	Mean Difference (I-J)	Std. Error	Sig.	95% Confidence Interval Lower Bound	95% Confidence Interval Upper Bound
0 ppm	50 ppm	-.0039500	.0086521	.653	-.022127	.014227
	75 ppm	-.0063750	.0086521	.471	-.024552	.011802
	100 ppm	-.0137250	.0086521	.130	-.031902	.004452
	150 ppm	-.0419250*	.0086521	.000	-.060102	-.023748
	200 ppm	-.0199750*	.0086521	.033	-.038152	-.001798
50 ppm	0 ppm	.0039500	.0086521	.653	-.014227	.022127
	75 ppm	-.0024250	.0086521	.782	-.020602	.015752
	100 ppm	-.0097750	.0086521	.273	-.027952	.008402
	150 ppm	-.0379750*	.0086521	.000	-.056152	-.019798
	200 ppm	-.0160250	.0086521	.080	-.034202	.002152
75 ppm	0 ppm	.0063750	.0086521	.471	-.011802	.024552
	50 ppm	.0024250	.0086521	.782	-.015752	.020602
	100 ppm	-.0073500	.0086521	.407	-.025527	.010827
	150 ppm	-.0355500*	.0086521	.001	-.053727	-.017373
	200 ppm	-.0136000	.0086521	.133	-.031777	.004577
100 ppm	0 ppm	.0137250	.0086521	.130	-.004452	.031902
	50 ppm	.0097750	.0086521	.273	-.008402	.027952
	75 ppm	.0073500	.0086521	.407	-.010827	.025527
	150 ppm	-.0282000*	.0086521	.004	-.046377	-.010023
	200 ppm	-.0062500	.0086521	.479	-.024427	.011927
150 ppm	0 ppm	.0419250*	.0086521	.000	.023748	.060102
	50 ppm	.0379750*	.0086521	.000	.019798	.056152
	75 ppm	.0355500*	.0086521	.001	.017373	.053727
	100 ppm	.0282000*	.0086521	.004	.010023	.046377
	200 ppm	.0219500*	.0086521	.021	.003773	.040127
200 ppm	0 ppm	.0199750*	.0086521	.033	.001798	.038152
	50 ppm	.0160250	.0086521	.080	-.002152	.034202
	75 ppm	.0136000	.0086521	.133	-.004577	.031777
	100 ppm	.0062500	.0086521	.479	-.011927	.024427
	150 ppm	-.0219500*	.0086521	.021	-.040127	-.003773

*. The mean difference is significant at the 0.05 level.

Perlakuan dolomit 150 berbeda nyata terhadap seluruh perlakuan

Duncan Test

Dry_Weight

Dolomite		N	Subset	
			1	2
Duncan[a]	0 ppm	4	.0319	
	50 ppm	4	.0358	
	75 ppm	4	.0383	
	100 ppm	4	.0456	
	200 ppm	4	.0519	
	150 ppm	4		.0738
	Sig.		.051	1.000

Means for groups in homogeneous subsets are displayed.
Based on observed means.
The error term is Mean Square(Error) = .000.

a. Uses Harmonic Mean Sample Size = 4.000.
