# Supplementary material for: Growth, secondary metabolite production, and in vitro antiplasmodial activity of Sonchus arvensis L. callus under dolomite [CaMg(CO3)2] treatment
Source: PLoS One. 2021 Aug 20;16(8):e0254804. doi: 10.1371/journal.pone.0254804 (PMC8378700; doi:10.1371/journal.pone.0254804)
Supplement: S2 Table — (PDF) [file pone.0254804.s002.pdf]

**Laboratorium PT. Gelora Djaja**

Abundance

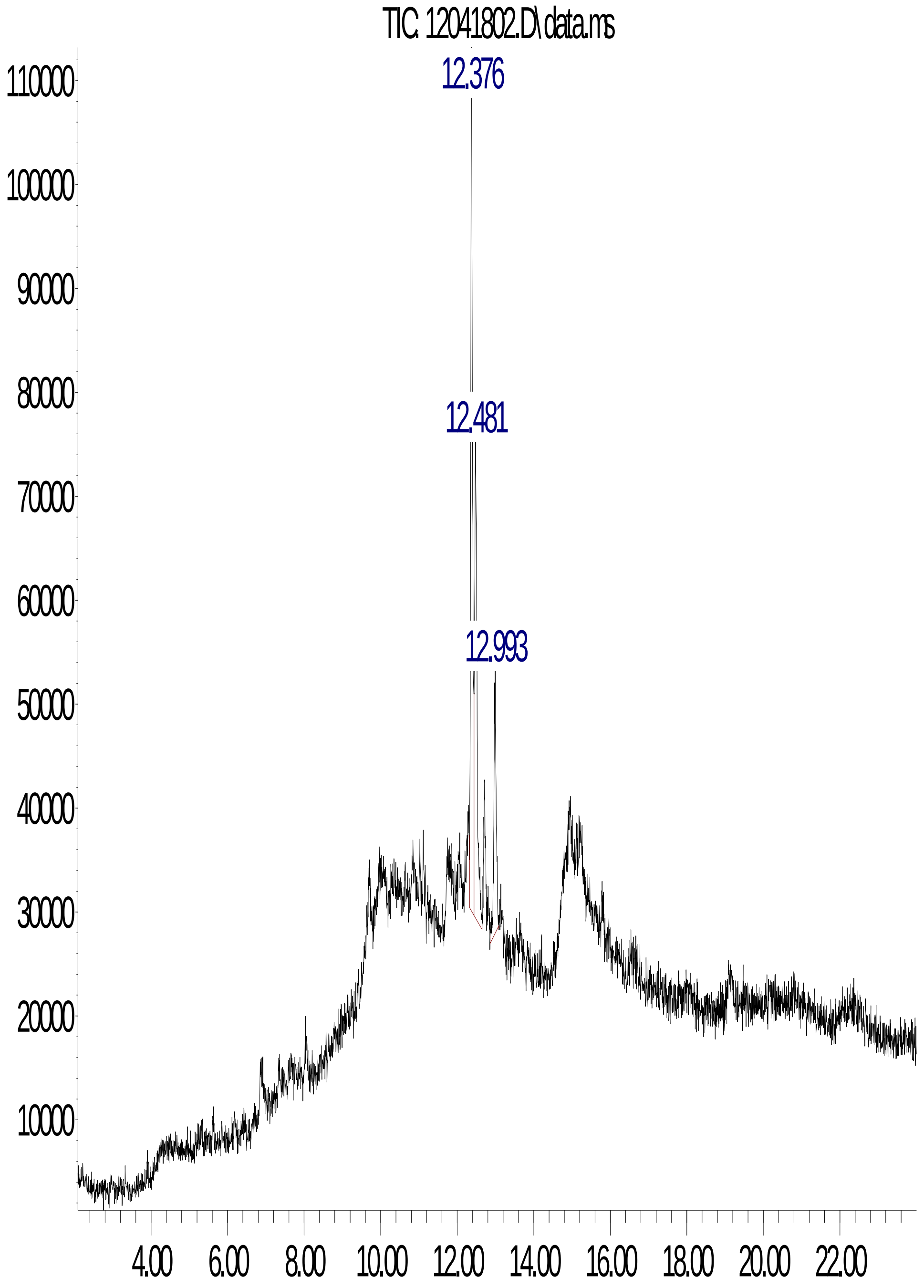


Time-->

# Laboratorium PT. Gelora Djaja

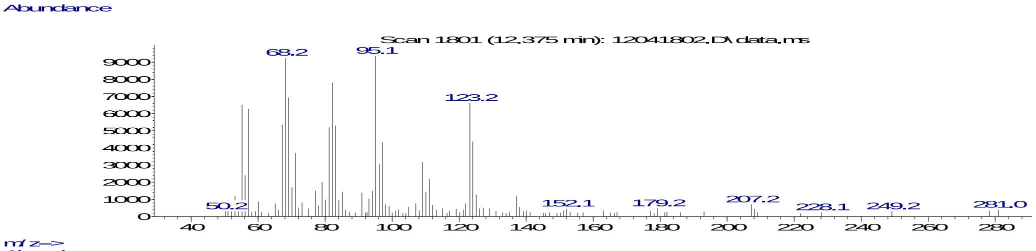


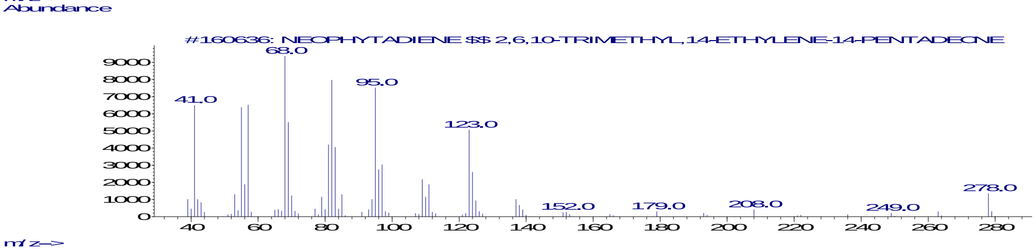


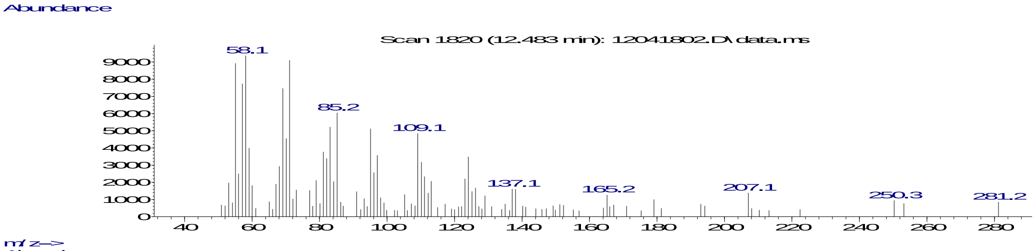


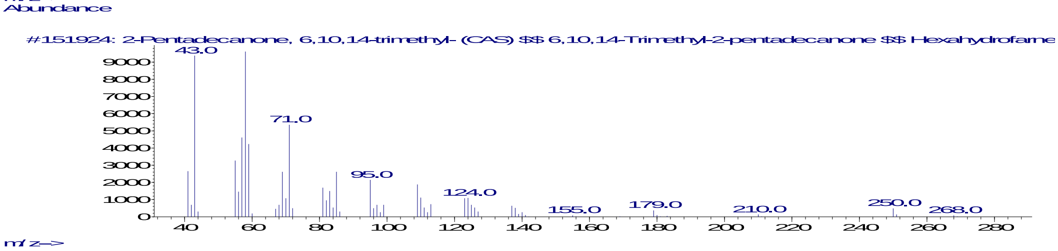


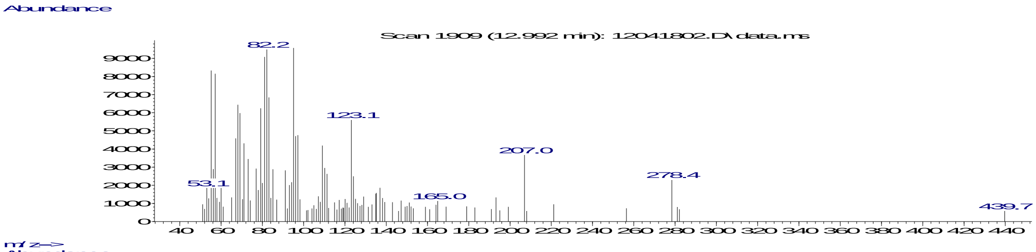


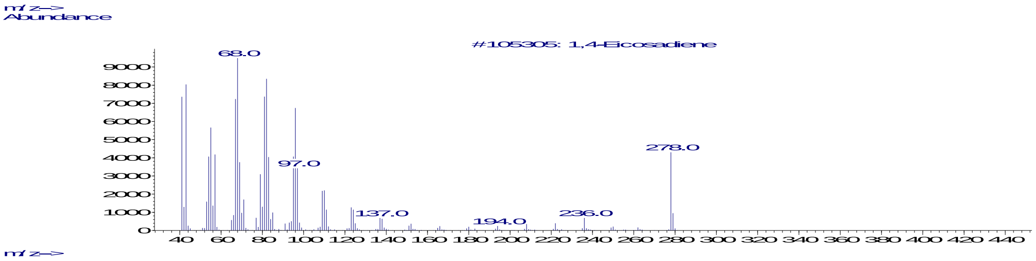

**Laboratorium PT. Gelora Djaja**

Library Search Report

Data Path : C:\msdchem\1\DATA\
Data File : 12041802.D
Acq On : 12 Apr 2018 13:33
Operator : SRA
Sample : 083 LU15 Daun + 1,5mL Ethanol
Misc : Shilfia N - UGM
ALS Vial : 2 Sample Multiplier: 1

Search Libraries: C:\Database\NIST02.L Minimum Quality: 85
C:\Database\Wiley275.L Minimum Quality: 85

Unknown Spectrum: Apex
Integration Events: Chemstation Integrator - autoint1.e

```
Pk#  RT  Area%     Library/ID             Ref#   CAS#   Qual
______________________________________________________________

 1  12.375 47.75 C:\Database\Wiley275.L
          NEOPHYTADIENE $$ 2,6,10-TRIMETHYL, 160636 000000-00-0 99
          14-ETHYLENE-14-PENTADECNE
          NEOPHYTADIENE $$ 2,6,10-TRIMETHYL, 160635 000000-00-0 98
          14-ETHYLENE-14-PENTADECNE
          2-Hexadecen-1-ol, 3,7,11,15-tetram 175359 000150-86-7 91
          ethyl-, [R-[R*,R*-(E)]]- (CAS) $$
          Phytol $$ trans-Phytol $$ (E)-(7R,
          11R)-3,7,11,15-tetramethyl-2-hexad
          ecen-1-ol $$ 2-Hexadecen-1-ol, 3,7
          ,11,15-tetramethyl-, [R-[R@,R@-(E)
          ]]- $$ 3,7,11,15-Tetramethyl-2-hex
          adecen-1-ol $$ (E

 2  12.483 35.33 C:\Database\Wiley275.L
          2-Pentadecanone, 6,10,14-trimethyl 151924 000502-69-2 46
          - (CAS) $$ 6,10,14-Trimethyl-2-pen
          tadecanone $$ Hexahydrofarnesyl ac
          etone
          14-.BETA.-H-PREGNA $$ 14-.BETA.-PR 169066 000000-00-0 35
          EGNA $$ 14B-PREGNANE
          11-Dodecen-2-one, 7,7-dimethyl- (C  96094 035194-22-0 35
          AS)
```

3 12.992 16.91 C:\Database\NIST02.L
1,4-Eicosadiene 105305 1000131-16-3 86
1,19-Eicosadiene 105307 014811-95-1 58
Cyclopentane, 1,2-dimethyl-3-(1-me 16400 006983-03-5 53
thylethenyl)-

EX-DAUN.M Tue Apr 17 13:08:35 2018

Surabaya, 17 April 2018

Mengetahui,

Penanggung jawab Pengujian,

Dr. Mohammad Holil
*Factory Lab. Manager*

Reo Dewa Kembara, S.Si
*Lab. Testing Technical Manager*
