# Supplementary material for: Growth, secondary metabolite production, and in vitro antiplasmodial activity of Sonchus arvensis L. callus under dolomite [CaMg(CO3)2] treatment
Source: PLoS One. 2021 Aug 20;16(8):e0254804. doi: 10.1371/journal.pone.0254804 (PMC8378700; doi:10.1371/journal.pone.0254804)
Supplement: S3 Table — (PDF) [file pone.0254804.s003.pdf]

Laboratorium PT. Gelora Djaja

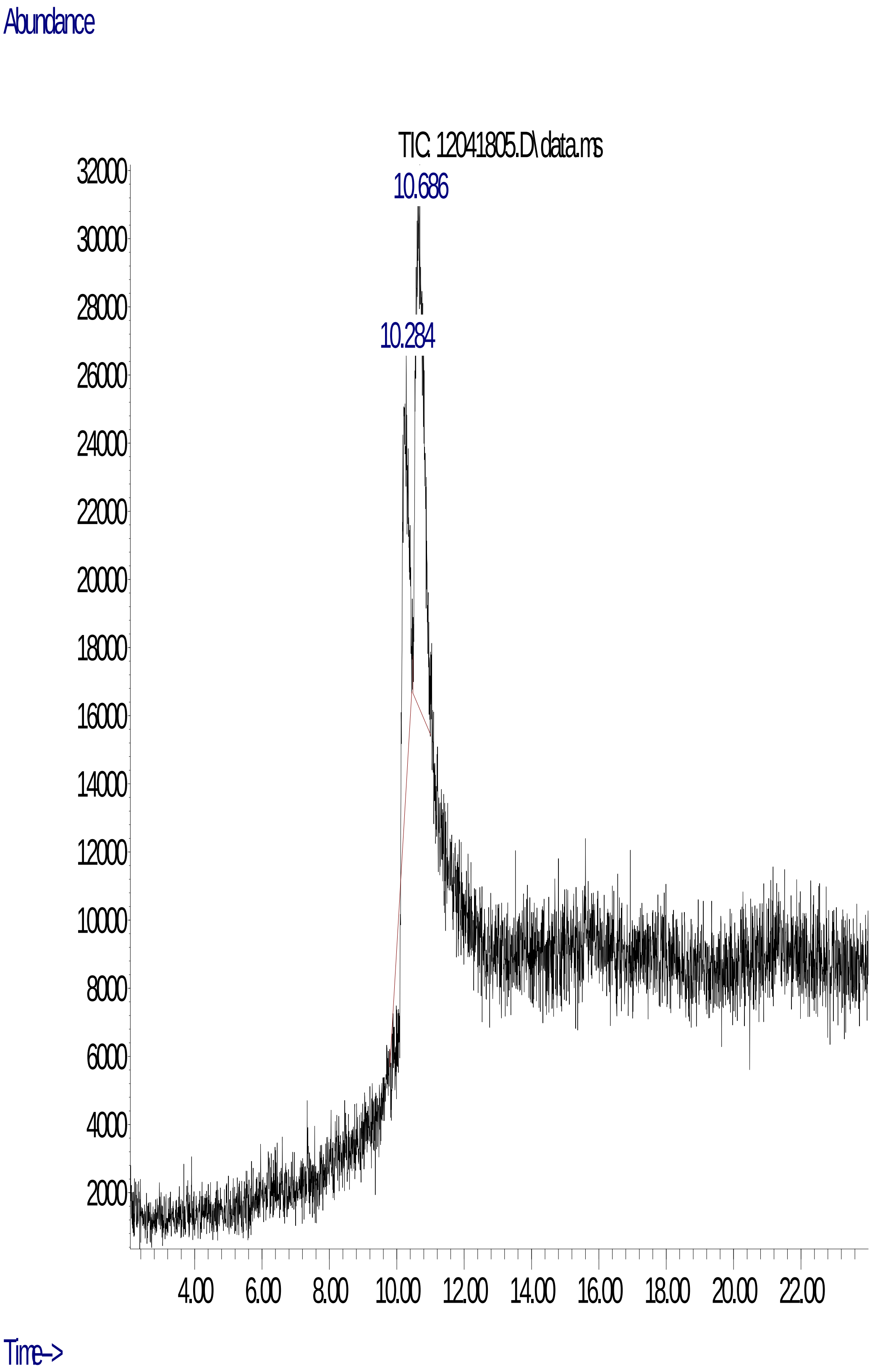

Laboratorium PT. Gelora Djaja

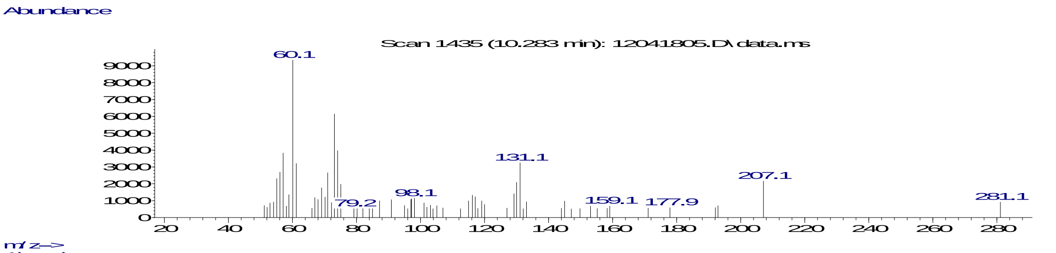

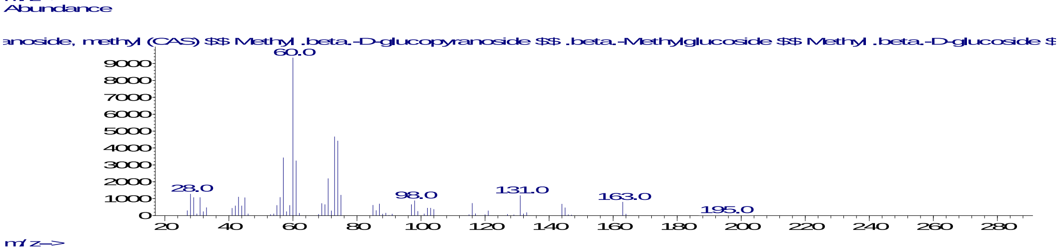

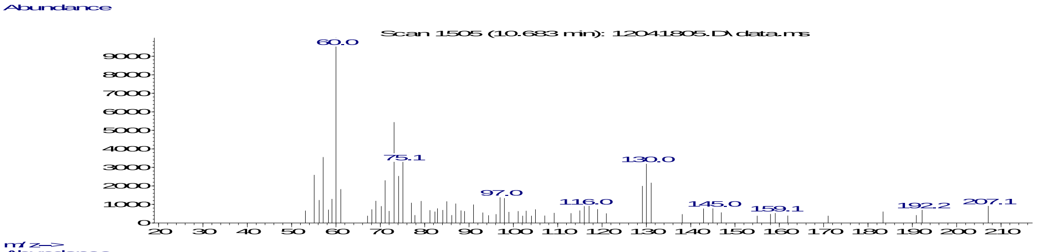

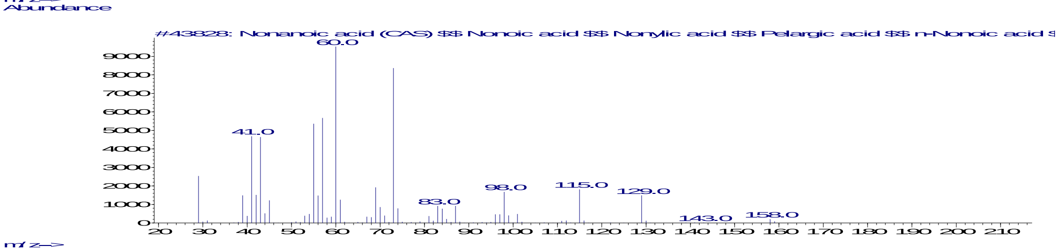

Laboratorium PT. Gelora Djaja

Library Search Report

Data Path : C:\msdchem\1\DATA\
Data File : 12041805.D
Acq On : 12 Apr 2018 14:58
Operator : SRA
Sample : 086 LU15 D1Bo15/kontrol + 1mL Ethanol
Misc : Shilfia N - UGM
ALS Vial : 5 Sample Multiplier: 1

Search Libraries: C:\Database\NIST02.L Minimum Quality: 85
C:\Database\Wiley275.L Minimum Quality: 85

Unknown Spectrum: Apex
Integration Events: Chemstation Integrator - autoint1.e

Pk#	RT	Area%	Library/ID	Ref#	CAS#	Qual
1	10.283	29.31	C:\Database\Wiley275.L			
			.beta.-D-Glucopyranoside, methyl (CAS) $$ Methyl .beta.-D-glucopyranoside $$ .beta.-Methylglucoside $$ Methyl .beta.-D-glucoside $$ Glucopyranoside, methyl, .beta.-D- $$ 1-O-Methyl-.beta.-D-glucopyranoside $$ .beta.-d-Methylglucopyranosid	77549	000709-50-2	47
			.alpha.-D-Galactopyranoside, methyl (CAS) $$ Methyl .alpha.-D-galactopyranoside $$ Methyl .alpha.-D-galactoside $$ .alpha.-Methyl-D-galactoside $$ .alpha.-Methyl-D-galactopyranoside $$ Galactopyranoside, methyl, .alpha.-D- $$ .alpha.-D-Galactopyranose met	77548	003396-99-4	47
			.BETA.-D-RIBOPYRANOSIDE, METHYL-	48342	000000-00-0	43
2	10.683	70.69	C:\Database\Wiley275.L			
			Nonanoic acid (CAS) $$ Nonoic acid $$ Nonylic acid $$ Pelargic acid $$ n-Nonoic acid $$ n-Nonylic acid $$ n-Nonanoic acid $$ 1-Octanecarboxylic acid $$ Pelargonic acid $$ Cirrasol 185a $$ Emfac 1202 $$ Hexacid C-9 $$ Pelargon $$ Pergonic acid	43828	000112-05-0	55

Nonanoic acid (CAS) $$ Nonoic acid $$ Nonylic acid $$ Pelargic acid $$ n-Nonoic acid $$ n-Nonylic acid $$ n-Nonanoic acid $$ 1-Octanecarboxylic acid $$ Pelargonic acid $$ Cirrasol 185a $$ Emfac 1202 $$ Hexacid C-9 $$ Pelargon $$ Pergonic acid	43826	000112-05-0	51
Nonanoic acid (CAS) $$ Nonoic acid $$ Nonylic acid $$ Pelargic acid $$ n-Nonoic acid $$ n-Nonylic acid $$ n-Nonanoic acid $$ 1-Octanecarboxylic acid $$ Pelargonic acid $$ Cirrasol 185a $$ Emfac 1202 $$ Hexacid C-9 $$ Pelargon $$ Pergonic acid	43827	000112-05-0	50

EX-DAUN.M Tue Apr 17 13:32:49 2018

Surabaya, 17 April 2018

Mengetahui, | Penanggung jawab Pengujian,

Dr. Mohammad Holil
Factory Lab. Manager

Reo Dewa Kembara, S.Si
Lab. Testing Technical Manager
