# Supplementary material for: Growth, secondary metabolite production, and in vitro antiplasmodial activity of Sonchus arvensis L. callus under dolomite [CaMg(CO3)2] treatment
Source: PLoS One. 2021 Aug 20;16(8):e0254804. doi: 10.1371/journal.pone.0254804 (PMC8378700; doi:10.1371/journal.pone.0254804)
Supplement: S4 Table — (PDF) [file pone.0254804.s004.pdf]

Laboratorium PT. Gelora Djaja

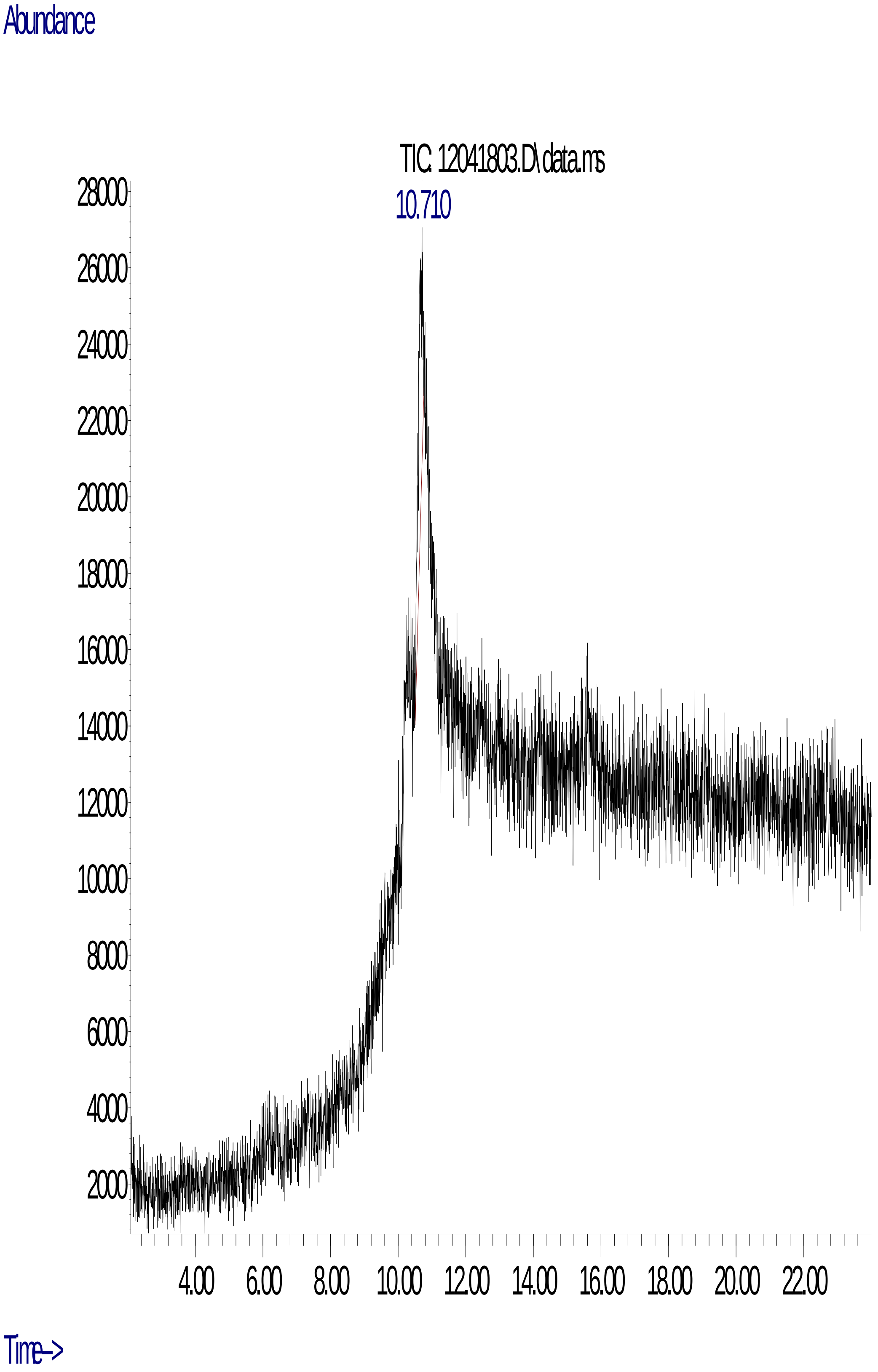

Laboratorium PT. Gelora Djaja

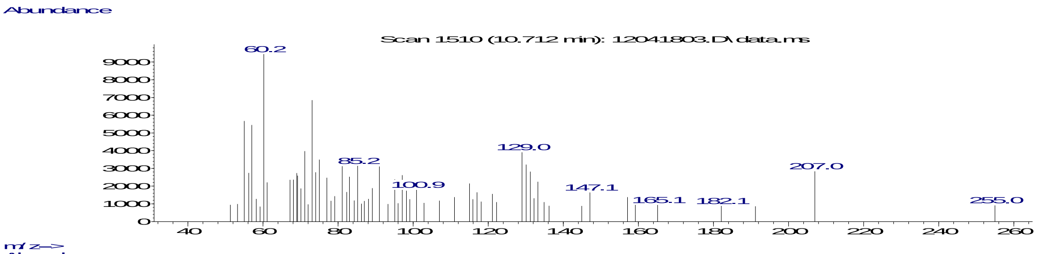

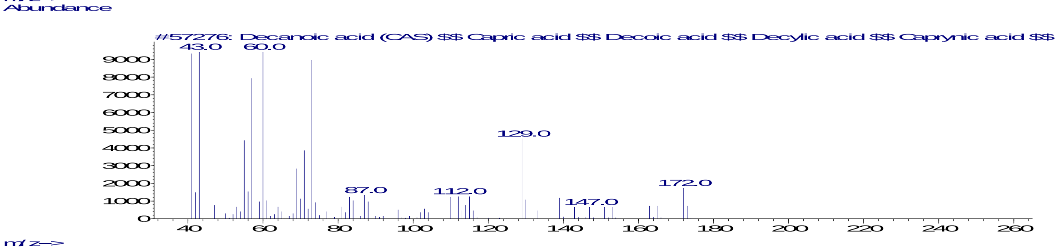

Laboratorium PT. Gelora Djaja

Library Search Report

Data Path : C:\msdchem\1\DATA\
Data File : 12041803.D
Acq On : 12 Apr 2018 14:01
Operator : SRA
Sample : 084 LU15 Dolomit 75 + 1mL Ethanol
Misc : Shilfia N - UGM
ALS Vial : 3 Sample Multiplier: 1

Search Libraries: C:\Database\NIST02.L Minimum Quality: 85
C:\Database\Wiley275.L Minimum Quality: 85

Unknown Spectrum: Apex
Integration Events: Chemstation Integrator - autoint1.e

Pk#	RT	Area%	Library/ID	Ref#	CAS#	Qual
1	10.712	100.00	C:\Database\Wiley275.L			
			Decanoic acid (CAS) $$ Capric acid $$ Decoic acid $$ Decylic acid $$ Caprynic acid $$ Caprinic acid $$ n-Decoic acid $$ n-Capric acid $$ n-Decylic acid $$ n-Decanoic acid $$ 1-Nonanecarboxylic acid $$ Emery 659 $$ Prifrac 296 $$ Nonane-1-carboxylic acid $	57276	000334-48-5	46
			Decanoic acid (CAS) $$ Capric acid $$ Decoic acid $$ Decylic acid $$ Caprynic acid $$ Caprinic acid $$ n-Decoic acid $$ n-Capric acid $$ n-Decylic acid $$ n-Decanoic acid $$ 1-Nonanecarboxylic acid $$ Emery 659 $$ Prifrac 296 $$ Nonane-1-carboxylic acid $	57270	000334-48-5	43
			decanoic acid $$ Capric acid	57486	000334-48-5	35

EX-DAUN.M Tue Apr 17 13:30:12 2018

Surabaya, 17 April 2018

Mengetahui,

Penanggung jawab Pengujian,

Dr. Mohammad Holil
Factory Lab. Manager

Reo Dewa Kembara, S.Si
Lab. Testing Technical Manager
