# Supplementary material for: Growth, secondary metabolite production, and in vitro antiplasmodial activity of Sonchus arvensis L. callus under dolomite [CaMg(CO3)2] treatment
Source: PLoS One. 2021 Aug 20;16(8):e0254804. doi: 10.1371/journal.pone.0254804 (PMC8378700; doi:10.1371/journal.pone.0254804)
Supplement: S5 Table — (PDF) [file pone.0254804.s005.pdf]

Laboratorium PT. Gelora Djaja

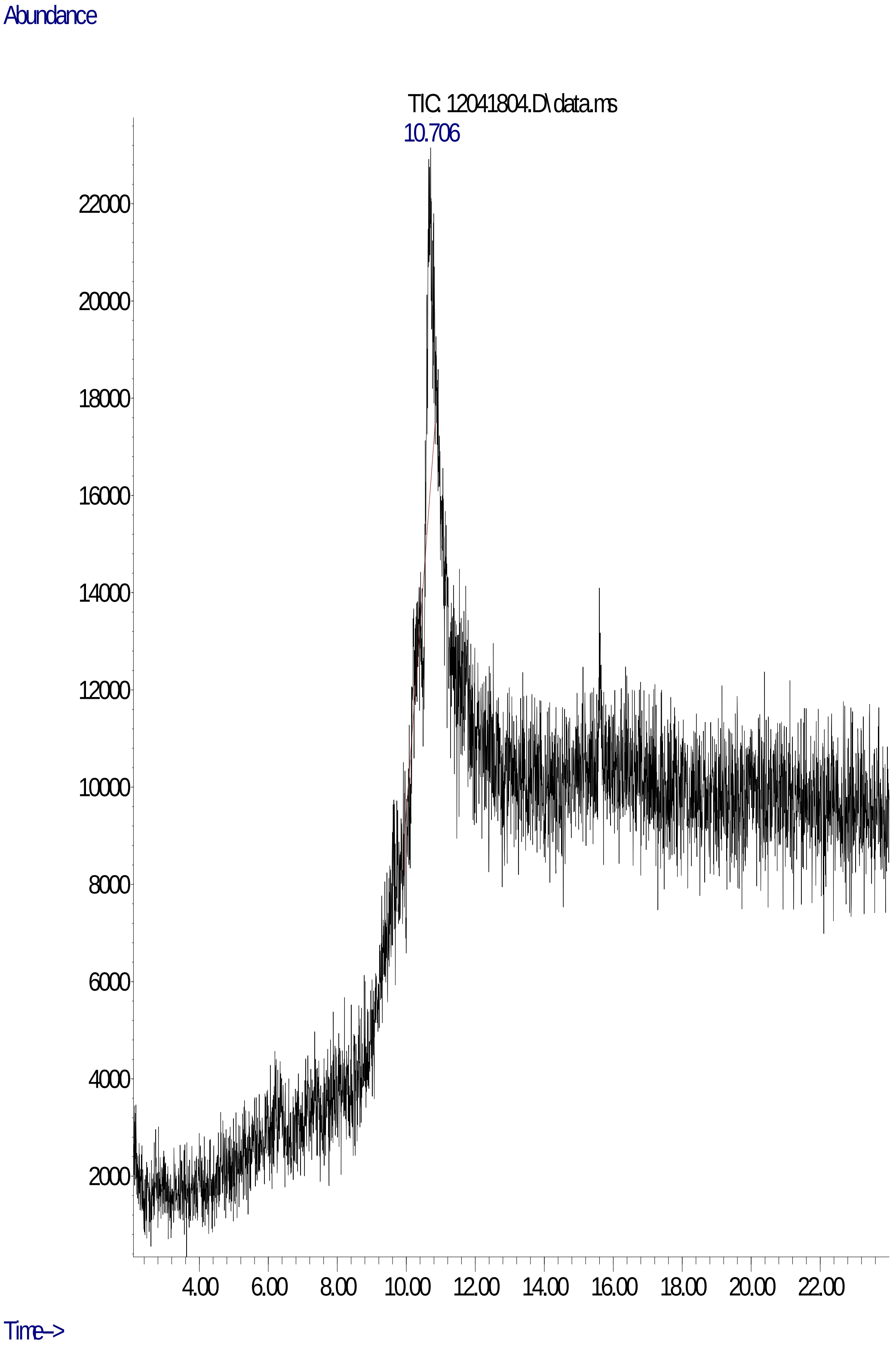

Laboratorium PT. Gelora Djaja

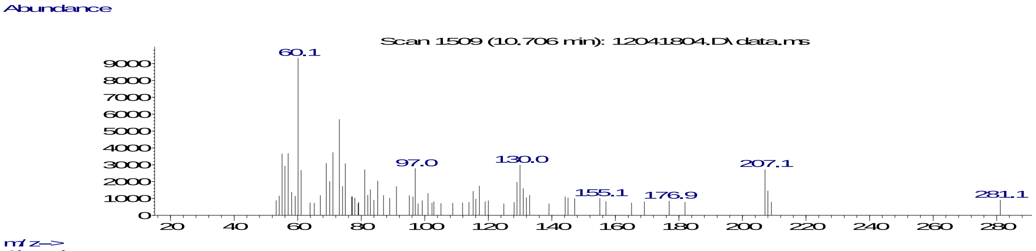

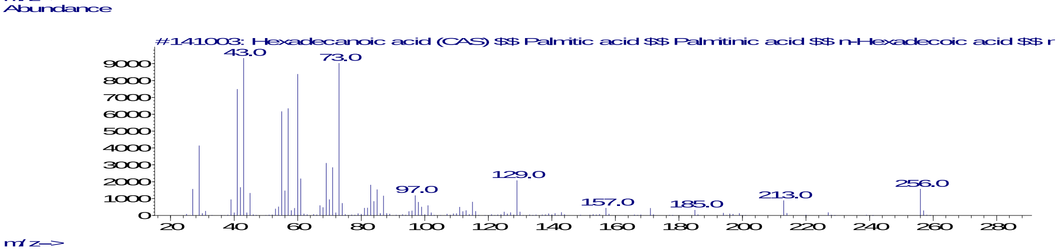

Laboratorium PT. Gelora Djaja

Library Search Report

Data Path : C:\msdchem\1\DATA\
Data File : 12041804.D
Acq On : 12 Apr 2018 14:30
Operator : SRA
Sample : 085 LU15 Dolomit 150 + 1mL Ethanol
Misc : Shilfia N - UGM
ALS Vial : 4 Sample Multiplier: 1

Search Libraries: C:\Database\NIST02.L Minimum Quality: 85
C:\Database\Wiley275.L Minimum Quality: 85

Unknown Spectrum: Apex
Integration Events: Chemstation Integrator - autoint1.e

Pk#	RT	Area%	Library/ID	Ref#	CAS#	Qual
1	10.706	100.00	C:\Database\Wiley275.L			
			Hexadecanoic acid (CAS) $$ Palmitic acid $$ Palmitinic acid $$ n-Hexadecoic acid $$ n-Hexadecanoic acid $$ Pentadecanecarboxylic acid $$ 1-Pentadecanecarboxylic acid $$ Prifrac 2960 $$ Coconut oil fatty acids $$ Cetylic acid $$ Emersol 140 $$ Emersol 143	141003	000057-10-3	50
			Tetradecanoic acid (CAS) $$ Myristic acid $$ MYRISTINIC ACID $$ n-Tetradecanoic acid $$ neo-Fat 14 $$ Univol U 316S $$ n-Tetradecoic acid $$ 1-Tridecanecarboxylic acid $$ n-Tetradecan-1-oic acid $$ methyl tridecanoate $$ Coconut oil fatty acids $$ Crodaci	114434	000544-63-8	47
			1H-Indole-2,3-dione, 5-methyl- (CAS) $$ 5-Methylisatin $$ Indole-2,3-dione, 5-methyl- $$ 5-Methylindole-2,3-dione $$ 2,3-Dihydro-5-methylindole-2,3-dione	46168	000608-05-9	43

EX-DAUN.M Tue Apr 17 13:31:35 2018

Laboratorium PT. Gelora Djaja

Surabaya, 17 April 2018

Mengetahui,

Dr. Mohammad Holil
Factory Lab. Manager

Penanggung jawab Pengujian,

Reo Dewa Kembara, S.Si
Lab. Testing Technical Manager
