# Supplementary material for: Growth, secondary metabolite production, and in vitro antiplasmodial activity of Sonchus arvensis L. callus under dolomite [CaMg(CO3)2] treatment
Source: PLoS One. 2021 Aug 20;16(8):e0254804. doi: 10.1371/journal.pone.0254804 (PMC8378700; doi:10.1371/journal.pone.0254804)
Supplement: S6 Table — (PDF) [file pone.0254804.s006.pdf]

ANTIMALARIA IN VITRO ASSAY AGAINTS *Plasmodium falciparum* 3D7

A. Leaves methanolic extract

Concentration (µg/mL)	Replication	% Parasitemia		Average of Growth Percentage (%)	Average of Inhibition Percentage (%)	IC_{50} (µg/mL)
		0 hour	48 hours			
Negative Control	1	1.23	3.68	3.07	-	17.78
	2	1.03	3.58			
	3	0.88	5.09			
	Average	1.05	4.12			
100	1	1.23	0.88	0	100	
	2	1.03	0.70			
	3	0.88	0.76			
	Average	1.05	0.78			
10	1	1.23	2.30	1.71	44.30	
	2	1.03	1.75			
	3	0.88	4.23			
	Average	1.05	2.76			
1	1	1.23	2.73	2.22	27.69	
	2	1.03	2.56			
	3	0.88	4.52			
	Average	1.05	3.27			
0.1	1	1.23	3.48	2.85	7.17	
	2	1.03	3.16			
	3	0.88	4.78			
	Average	1.05	3.90			
0.01	1	1.23	3.66	2.90	5.54	
	2	1.03	3.35			
	3	0.88	4.84			
	Average	1.05	3.95			

Confidence Limits

	Probability	95% Confidence Limits for dosis			95% Confidence Limits for log(dosis)[a]		
		Estimate	Lower Bound	Upper Bound	Estimate	Lower Bound	Upper Bound
PROBIT	.010	.001	.000	.004	-3.053	-4.212	-2.369
	.020	.003	.000	.011	-2.548	-3.528	-1.964
	.030	.006	.001	.020	-2.229	-3.097	-1.705
	.040	.010	.002	.031	-1.988	-2.773	-1.509
	.050	.016	.003	.045	-1.792	-2.511	-1.348
	.060	.024	.005	.062	-1.626	-2.289	-1.210
	.070	.033	.008	.082	-1.480	-2.096	-1.088
	.080	.045	.012	.105	-1.349	-1.924	-.978
	.090	.059	.017	.133	-1.230	-1.768	-.877
	.100	.076	.024	.165	-1.120	-1.626	-.783
	.150	.215	.089	.418	-.667	-1.051	-.378
	.200	.494	.239	.932	-.307	-.621	-.030
	.250	1.006	.525	1.975	.002	-.279	.296
	.300	1.906	1.008	4.095	.280	.003	.612
	.350	3.446	1.771	8.372	.537	.248	.923
	.400	6.044	2.945	16.943	.781	.469	1.229
	.450	10.411	4.737	34.089	1.017	.675	1.533
	.500	**17.779**	7.474	68.611	1.250	.874	1.836
	.550	30.360	11.700	139.199	1.482	1.068	2.144
	.600	52.294	18.340	287.323	1.718	1.263	2.458
	.650	91.734	29.055	610.423	1.963	1.463	2.786
	.700	165.864	47.013	1355.502	2.220	1.672	3.132
	.750	314.293	78.777	3216.241	2.497	1.896	3.507
	.800	640.380	139.576	8441.754	2.806	2.145	3.926
	.850	1468.027	271.122	26069.199	3.167	2.433	4.416
	.900	4169.359	623.242	108043.251	3.620	2.795	5.034
	.910	5364.924	761.723	152372.382	3.730	2.882	5.183
	.920	7055.278	947.109	221397.568	3.849	2.976	5.345
	.930	9534.692	1203.238	333935.909	3.979	3.080	5.524
	.940	13346.933	1571.705	528551.278	4.125	3.196	5.723
	.950	19587.569	2131.085	892529.126	4.292	3.329	5.951
	.960	30740.646	3046.790	1652182.217	4.488	3.484	6.218
	.970	53498.447	4726.594	3523549.428	4.728	3.675	6.547
	.980	111741.478	8469.286	9647992.676	5.048	3.928	6.984
	.990	356758.415	21216.620	47241435.225	5.552	4.327	7.674

a. Logarithm base = 10.

B. Leaves ethanolic extrat

Concentration (μg/mL)	Replication	% Parasitemia		Average of Growth Percentage (%)	Average of Inhibition Percentage (%)	IC_{50} (μg/mL)
		0 hour	48 hours			
Negative Control	1	1.23	4.15	2.78	-	27.09
	2	1.03	3.96			
	3	0.88	3.39			
	Average	1.05	3.83			
100	1	1.23	0.74	0	100	
	2	1.03	0.57			
	3	0.88	0.57			
	Average	1.05	0.63			
10	1	1.23	2.63	1.76	36.69	
	2	1.03	2.46			
	3	0.88	3.33			
	Average	1.05	2.81			
1	1	1.23	2.76	1.90	31.65	
	2	1.03	2.67			
	3	0.88	3.43			
	Average	1.05	2.95			
0.1	1	1.23	3.70	2.59	6.83	
	2	1.03	3.59			
	3	0.88	3.64			
	Average	1.05	3.64			
0.01	1	1.23	4.22	3.07	0	
	2	1.03	4.07			
	3	0.88	4.08			
	Average	1.05	4.12			

Confidence Limits

	Probability	95% Confidence Limits for dosis			95% Confidence Limits for log(dosis)[b]		
		Estimate	Lower Bound	Upper Bound	Estimate	Lower Bound	Upper Bound
PROBIT[a]	.010	.001	.	.	-3.103	.	.
	.020	.003	.	.	-2.571	.	.
	.030	.006	.	.	-2.234	.	.
	.040	.010	.	.	-1.981	.	.
	.050	.017	.	.	-1.774	.	.
	.060	.025	.	.	-1.599	.	.
	.070	.036	.	.	-1.445	.	.
	.080	.049	.	.	-1.307	.	.
	.090	.066	.	.	-1.182	.	.
	.100	.086	.	.	-1.066	.	.
	.150	.258	.	.	-.588	.	.
	.200	.619	.	.	-.209	.	.
	.250	1.310	.	.	.117	.	.
	.300	2.569	.	.	.410	.	.
	.350	4.795	.	.	.681	.	.
	.400	8.670	.	.	.938	.	.
	.450	15.378	.	.	1.187	.	.
	.500	**27.029**	.	.	1.432	.	.
	.550	47.507	.	.	1.677	.	.
	.600	84.264	.	.	1.926	.	.
	.650	152.362	.	.	2.183	.	.
	.700	284.422	.	.	2.454	.	.
	.750	557.840	.	.	2.747	.	.
	.800	1181.064	.	.	3.072	.	.
	.850	2831.333	.	.	3.452	.	.
	.900	8506.729	.	.	3.930	.	.
	.910	11095.807	.	.	4.045	.	.
	.920	14808.850	.	.	4.171	.	.
	.930	20340.613	.	.	4.308	.	.
	.940	28994.307	.	.	4.462	.	.
	.950	43440.222	.	.	4.638	.	.
	.960	69851.401	.	.	4.844	.	.
	.970	125248.909	.	.	5.098	.	.
	.980	272200.810	.	.	5.435	.	.
	.990	925177.592	.	.	5.966	.	.

a. A heterogeneity factor is used.

b. Logarithm base = 10.

C. 2,4-D 1 mg/L + BAP 0,5 mg/L callus methanolic extract

Concentration (µg/mL)	R	% Parasitemia		Average of Growth Percentage (%)	Average of Growth Percentage (%)	IC_{50} (µg/mL)
		0 hour	48 hours			
Neagtive Control	1	0.66	2.43	2.55	-	12.469
	2	0.90	2.58			
	3	1.00	5.19			
	Average	0.85	3.40			
100	1	0.66	0.97	0.41	83.92	
	2	0.90	1.16			
	3	1.00	1.65			
	Average	0.85	1.26			
10	1	0.66	1.45	1.64	35.69	
	2	0.90	2.20			
	3	1.00	3.81			
	Average	0.85	2.49			
1	1	0.66	2.09	2.11	17.25	
	2	0.90	2.40			
	3	1.00	4.38			
	Average	0.85	2.96			
0.1	1	0.66	2.23	2.32	9.02	
	2	0.90	2.70			
	3	1.00	4.58			
	Average	0.85	3.17			
0.01	1	0.66	2.60	2.61	0	
	2	0.90	2.83			
	3	1.00	4.95			
	Average	0.85	3.46			

Confidence Limits

		95% Confidence Limits for dosis			95% Confidence Limits for log(dosis)[b]		
	Probability	Estimate	Lower Bound	Upper Bound	Estimate	Lower Bound	Upper Bound
PROBIT[a]	.010	.013	.000	.419	-1.872	-349.226	-.378
	.020	.030	.000	.669	-1.524	-302.788	-.175
	.030	.050	.000	.911	-1.304	-273.330	-.040
	.040	.073	.000	1.159	-1.138	-251.173	.064
	.050	.099	.000	1.420	-1.003	-233.153	.152
	.060	.130	.000	1.697	-.888	-217.818	.230
	.070	.163	.000	1.996	-.787	-204.375	.300
	.080	.201	.000	2.321	-.697	-192.340	.366
	.090	.243	.000	2.676	-.615	-181.398	.428
	.100	.289	.000	3.067	-.539	-171.327	.487
	.150	.594	.000	5.847	-.226	-129.668	.767
	.200	1.052	.000	11.596	.022	-96.633	1.064
	.250	1.719	.000	28.140	.235	-68.423	1.449
	.300	2.672	.000	122.984	.427	-43.383	2.090
	.350	4.020	.000	4996.866	.604	-21.195	3.699
	.400	5.924	.000	73652523506.268	.773	-5.783	10.867
	.450	8.620	.028	3034719704173050500000000000 0.000	.936	-1.552	28.482
	.500	**12.469**	.349	7.732E+48	1.096	-.457	48.888
	.550	18.036	1.101	7.756E+69	1.256	.042	69.890
	.600	26.244	2.247	2.671E+91	1.419	.352	91.427
	.650	38.672	3.810	5.997E+113	1.587	.581	113.778
	.700	58.186	5.908	2.420E+137	1.765	.771	137.384
	.750	90.426	8.789	7.787E+162	1.956	.944	162.891
	.800	147.743	12.943	2.086E+191	2.170	1.112	191.319
	.850	261.837	19.425	2.985E+224	2.418	1.288	224.475
	.900	537.940	31.043	1.625E+266	2.731	1.492	266.211
	.910	640.120	34.595	1.965E+276	2.806	1.539	276.293
	.920	773.235	38.851	1.768E+287	2.888	1.589	287.247
	.930	951.765	44.059	1.963E+299	2.979	1.644	299.293
	.940	1200.299	50.604	.	3.079	1.704	312.746
	.950	1563.891	59.134	.	3.194	1.772	328.091
	.960	2134.122	70.824	.	3.329	1.850	346.121
	.970	3127.521	88.120	.	3.495	1.945	368.287
	.980	5198.081	117.278	.	3.716	2.069	397.755
	.990	11577.197	182.500	.	4.064	2.261	444.205

a. A heterogeneity factor is used.
b. Logarithm base = 10.

D. **2,4-D 1 mg/L + BAP 0,5 mg/L callus ethanolic extract**

Concentration (µg/mL)	Replication	% Parasitemia		Average of Growth Percentage (%)	Average of Growth Percentage (%)	IC_{50} (µg/mL)
		0 hour	48 hours			
Negative Control	1	0.66	2.60	2.58	-	> 10
	2	0.90	2.72			
	3	1.00	4.97			
	Average	0.85	3.43			
100	1	0.66	0.43	0	100	
	2	0.90	0.58			
	3	1.00	1.23			
	Average	0.85	0.75			
10	1	0.66	2.51	2.33	9.69	
	2	0.90	2.61			
	3	1.00	4.42			
	Average	0.85	3.18			
1	1	0.66	2.67	2.60	0	
	2	0.90	2.76			
	3	1.00	4.91			
	Average	0.85	3.45			
0.1	1	0.66	2.88	2.76	0	
	2	0.90	2.92			
	3	1.00	5.04			
	Average	0.85	3.61			
0.01	1	0.66	3.08	3.06	0	
	2	0.90	3.38			
	3	1.00	5.26			
	Average	0.85	3.91			

Confidence Limits

	Probability	95% Confidence Limits for concentration			95% Confidence Limits for log(concentration)[a]		
		Estimate	Lower Bound	Upper Bound	Estimate	Lower Bound	Upper Bound
PROBIT	.010	4.347	2.414	6.481	.638	.383	.812
	.020	5.283	3.083	7.663	.723	.489	.884
	.030	5.979	3.598	8.530	.777	.556	.931
	.040	6.562	4.038	9.251	.817	.606	.966
	.050	7.078	4.434	9.887	.850	.647	.995
	.060	7.549	4.800	10.466	.878	.681	1.020
	.070	7.988	5.144	11.005	.902	.711	1.042
	.080	8.402	5.472	11.513	.924	.738	1.061
	.090	8.798	5.786	11.998	.944	.762	1.079
	.100	9.178	6.091	12.465	.963	.785	1.096
	.150	10.937	7.518	14.630	1.039	.876	1.165
	.200	12.572	8.863	16.661	1.099	.948	1.222
	.250	14.169	10.183	18.670	1.151	1.008	1.271
	.300	15.774	11.512	20.722	1.198	1.061	1.316
	.350	17.424	12.873	22.867	1.241	1.110	1.359
	.400	19.149	14.287	25.154	1.282	1.155	1.401
	.450	20.980	15.775	27.632	1.322	1.198	1.441
	.500	22.953	17.360	30.361	1.361	1.240	1.482
	.550	25.111	19.072	33.419	1.400	1.280	1.524
	.600	27.512	20.948	36.906	1.440	1.321	1.567
	.650	30.236	23.038	40.967	1.481	1.362	1.612
	.700	33.398	25.419	45.818	1.524	1.405	1.661
	.750	37.182	28.208	51.805	1.570	1.450	1.714
	.800	41.904	31.604	59.532	1.622	1.500	1.775
	.850	48.169	35.986	70.190	1.683	1.556	1.846
	.900	57.399	42.227	86.649	1.759	1.626	1.938
	.910	59.881	43.869	91.216	1.777	1.642	1.960
	.920	62.700	45.715	96.471	1.797	1.660	1.984
	.930	65.953	47.825	102.621	1.819	1.680	2.011
	.940	69.786	50.284	109.982	1.844	1.701	2.041
	.950	74.430	53.227	119.061	1.872	1.726	2.076
	.960	80.283	56.883	130.739	1.905	1.755	2.116

	.970	88.112	61.693	146.751	1.945	1.790	2.167
	.980	99.714	68.670	171.245	1.999	1.837	2.234
	.990	121.180	81.181	218.736	2.083	1.909	2.340

a. Logarithm base = 10.

E. Callus dolomite (150mg/L) methanolic extract

Concentration (µg/mL)	Replication	% Parasitemia		Average of Growth Percentage (%)	Average of Growth Percentage (%)	IC_{50} (µg/mL)
		0 hour	48 hours			
Negative Control	1	1.23	3.53	2.91	--	5.037
	2	1.03	3.48			
	3	0.88	4.87			
	Average	1.05	3.96			
100	1	1.23	0.91	0.10	96.56	
	2	1.03	0.83			
	3	0.88	1.71			
	Average	1.05	1.15			
10	1	1.23	2.23	1.34	53.95	
	2	1.03	1.79			
	3	0.88	3.14			
	Average	1.05	2.39			
1	1	1.23	3.57	2.39	17.87	
	2	1.03	3.35			
	3	0.88	3.41			
	Average	1.05	3.44			
0.1	1	1.23	3.75	2.64	9.28	
	2	1.03	3.57			
	3	0.88	3.75			
	Average	1.05	3.69			
0.01	1	1.23	4.19	3.02	0	
	2	1.03	3.95			
	3	0.88	4.08			
	Average	1.05	4.07			

Confidence Limits

	Probability	95% Confidence Limits for dosis			95% Confidence Limits for log(dosis)[b]		
		Estimate	Lower Bound	Upper Bound	Estimate	Lower Bound	Upper Bound
PROBIT[a]	.010	.025	.000	.462	-1.601	-54.976	-.335
	.020	.047	.000	.675	-1.331	-48.182	-.171
	.030	.069	.000	.867	-1.160	-43.875	-.062
	.040	.093	.000	1.053	-1.031	-40.639	.023
	.050	.118	.000	1.241	-.927	-38.008	.094
	.060	.145	.000	1.433	-.837	-35.771	.156
	.070	.174	.000	1.633	-.759	-33.812	.213
	.080	.205	.000	1.842	-.689	-32.059	.265
	.090	.237	.000	2.064	-.625	-30.467	.315
	.100	.271	.000	2.300	-.567	-29.002	.362
	.150	.474	.000	3.786	-.324	-22.962	.578
	.200	.739	.000	6.188	-.131	-18.202	.792
	.250	1.082	.000	10.741	.034	-14.175	1.031
	.300	1.524	.000	21.561	.183	-10.647	1.334
	.350	2.092	.000	58.481	.321	-7.530	1.767
	.400	2.827	.000	294.290	.451	-4.863	2.469
	.450	3.782	.002	4735.880	.578	-2.810	3.675
	.500	**5.037**	.032	363986.491	.702	-1.488	5.561
	.550	6.708	.187	101834202.589	.827	-.727	8.008
	.600	8.974	.536	64631870086.250	.953	-.270	10.810
	.650	12.125	1.088	74519328744130.330	1.084	.037	13.872
	.700	16.650	1.851	155431186342863008.000	1.221	.267	17.192
	.750	23.444	2.878	677990329787021800000.000	1.370	.459	20.831
	.800	34.318	4.289	8398902808416068000000000.000	1.536	.632	24.924
	.850	53.509	6.356	532399046225334550000000000000.000	1.728	.803	29.726
	.900	93.571	9.780	625337676123361340000000000000000000.000	1.971	.990	35.796
	.910	107.094	10.775	1.842E+37	2.030	1.032	37.265
	.920	124.009	11.942	7.285E+38	2.093	1.077	38.862
	.930	145.707	13.338	4.165E+40	2.163	1.125	40.620
	.940	174.457	15.048	3.832E+42	2.242	1.177	42.583
	.950	214.233	17.215	6.674E+44	2.331	1.236	44.824
	.960	272.698	20.089	2.877E+47	2.436	1.303	47.459
	.970	366.873	24.180	5.007E+50	2.565	1.383	50.700
	.980	544.223	30.742	1.024E+55	2.736	1.488	55.010
	.990	1013.226	44.389	6.445E+61	3.006	1.647	61.809

a. A heterogeneity factor is used.

b. Logarithm base = 10.

F. Callus dolomite (150mg/L) ethanolic extract

Concentration (μg/mL)	Replication	% Parasitemia		Average of Growth Percentage (%)	Average of Growth Percentage (%)	IC_{50} (μg/mL)
		0 hour	48 hours			
Negative Control	1	1.23	4.09	2.78	-	5.944
	2	1.03	3.98			
	3	0.88	3.41			
	Average	1.05	3.83			
100	1	1.23	0.86	0	100	
	2	1.03	0.76			
	3	0.88	0.39			
	Average	1.05	0.67			
10	1	1.23	2.27	1.11	60.07	
	2	1.03	1.93			
	3	0.88	2.28			
	Average	1.05	2.16			
1	1	1.23	3.69	2.18	21.58	
	2	1.03	3.44			
	3	0.88	2.56			
	Average	1.05	3.23			
0.1	1	1.23	3.94	2.46	11.51	
	2	1.03	3.85			
	3	0.88	2.74			
	Average	1.05	3.51			
0.01	1	1.23	4.14	2.84	0	
	2	1.03	4.04			
	3	0.88	3.48			
	Average	1.05	3.89			

Confidence Limits

	Probability	95% Confidence Limits for dosis			95% Confidence Limits for log(dosis)[b]		
		Estimate	Lower Bound	Upper Bound	Estimate	Lower Bound	Upper Bound
PROBIT[a]	.010	.005	.	.	-2.288	.	.
	.020	.012	.	.	-1.929	.	.
	.030	.020	.	.	-1.701	.	.
	.040	.030	.	.	-1.530	.	.
	.050	.041	.	.	-1.391	.	.
	.060	.053	.	.	-1.272	.	.
	.070	.068	.	.	-1.168	.	.
	.080	.084	.	.	-1.075	.	.
	.090	.102	.	.	-.991	.	.
	.100	.122	.	.	-.913	.	.
	.150	.257	.	.	-.590	.	.
	.200	.464	.	.	-.334	.	.
	.250	.770	.	.	-.114	.	.
	.300	1.213	.	.	.084	.	.
	.350	1.849	.	.	.267	.	.
	.400	2.758	.	.	.441	.	.
	.450	4.061	.	.	.609	.	.
	.500	**5.944**	.	.	.774	.	.
	.550	8.699	.	.	.939	.	.
	.600	12.809	.	.	1.108	.	.
	.650	19.109	.	.	1.281	.	.
	.700	29.126	.	.	1.464	.	.
	.750	45.902	.	.	1.662	.	.
	.800	76.174	.	.	1.882	.	.
	.850	137.472	.	.	2.138	.	.
	.900	288.959	.	.	2.461	.	.
	.910	345.747	.	.	2.539	.	.
	.920	420.156	.	.	2.623	.	.
	.930	520.584	.	.	2.716	.	.
	.940	661.373	.	.	2.820	.	.
	.950	868.978	.	.	2.939	.	.
	.960	1197.579	.	.	3.078	.	.
	.970	1776.438	.	.	3.250	.	.
	.980	3000.485	.	.	3.477	.	.
	.990	6854.609	.	.	3.836	.	.

a. A heterogeneity factor is used.
b. Logarithm base = 10.
