# Supplementary material for: Growth, secondary metabolite production, and in vitro antiplasmodial activity of Sonchus arvensis L. callus under dolomite [CaMg(CO3)2] treatment
Source: PLoS One. 2021 Aug 20;16(8):e0254804. doi: 10.1371/journal.pone.0254804 (PMC8378700; doi:10.1371/journal.pone.0254804)

Fig.1. Morphology of *Sonchus arvensis* L. callus on media with 1mg/L 2,4D + 0,5 mg/L BAP

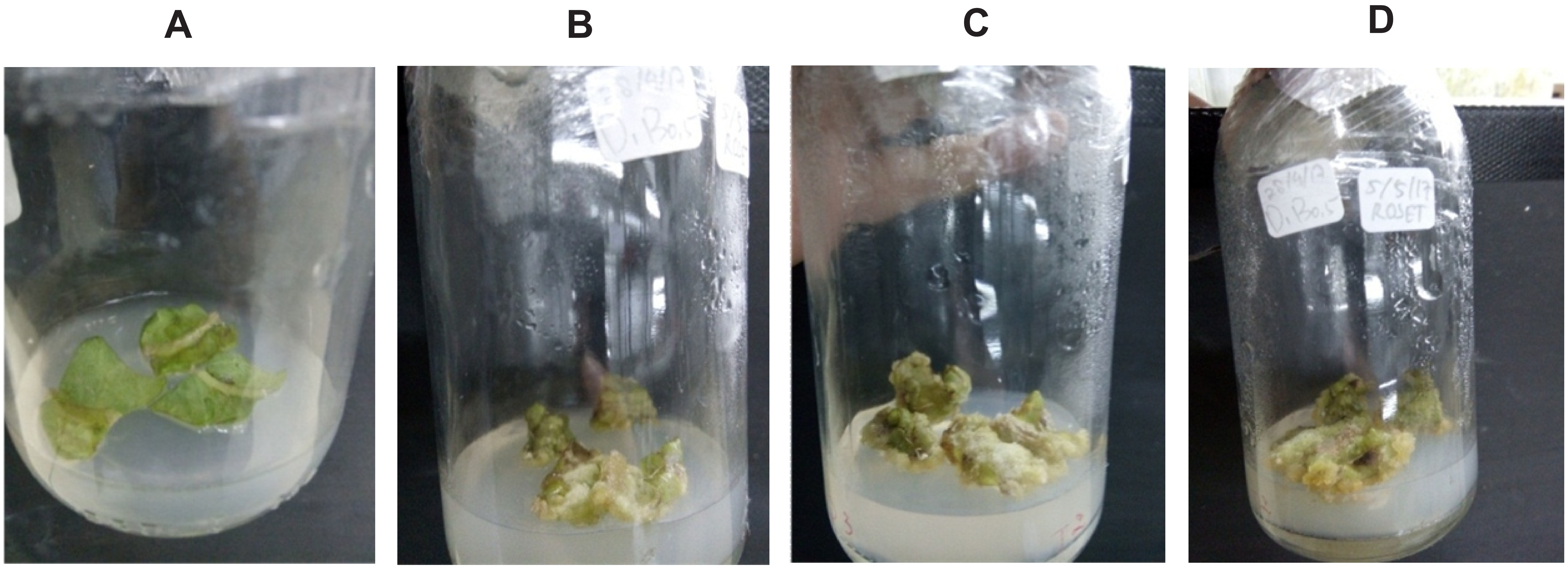

Fig.2. Morphology of *Sonchus arvensis* L. callus on dolomite media

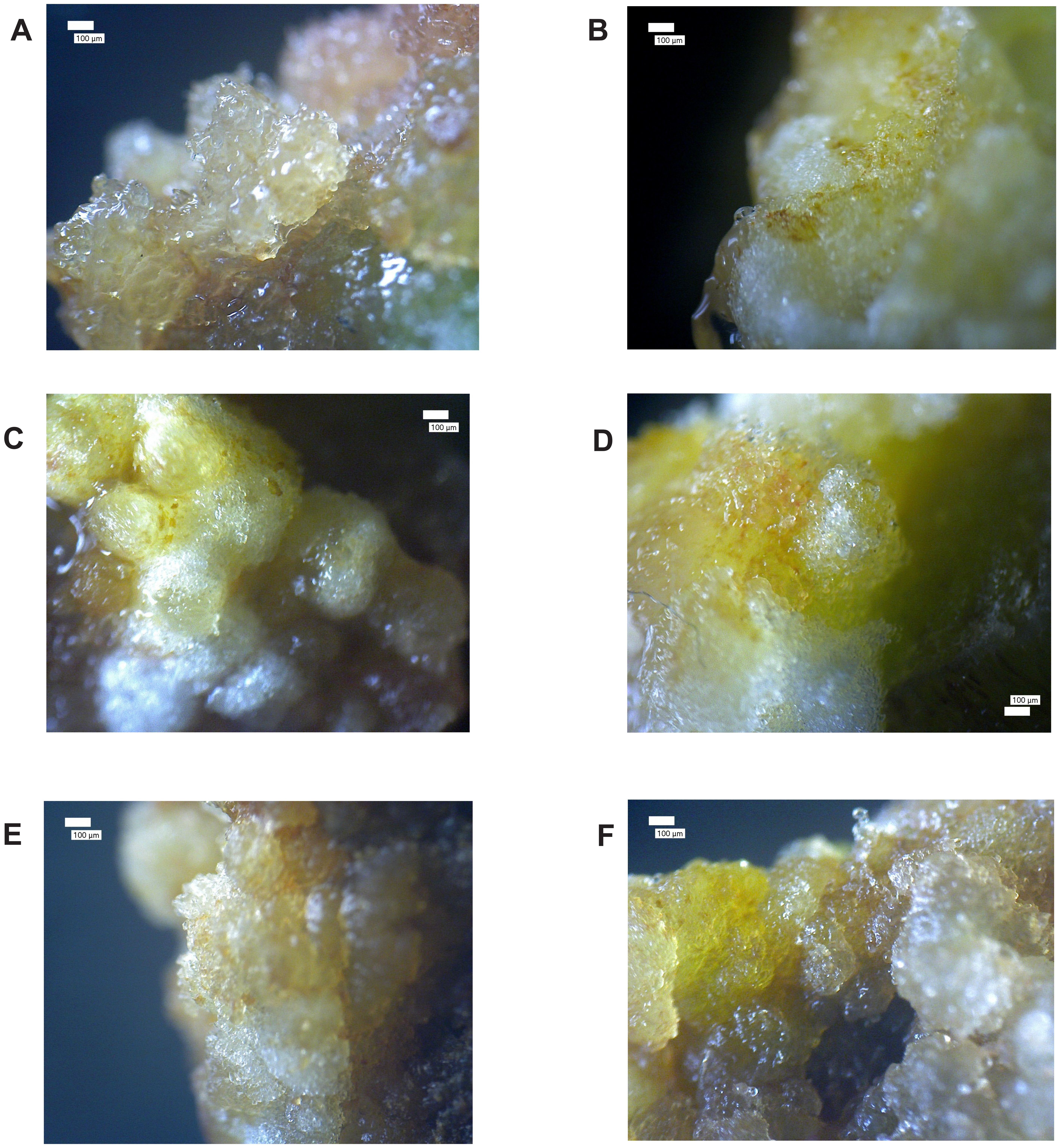

Fig.3. Embryogenic of *Sonchus arvensis* L. callus on dolomite media

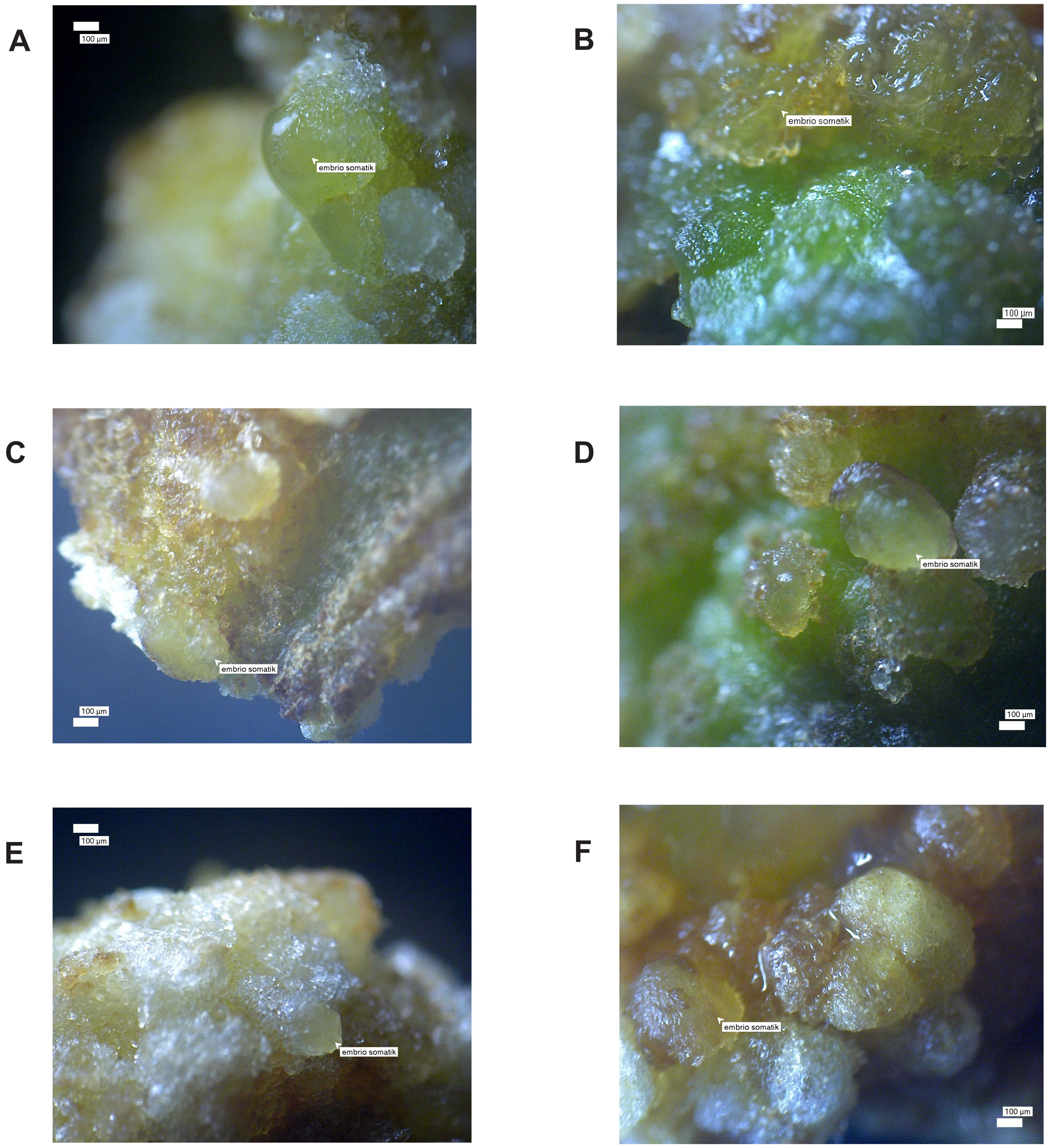

Fig.4. Cross-section of *Sonchus arvensis* L. callus on dolomite media

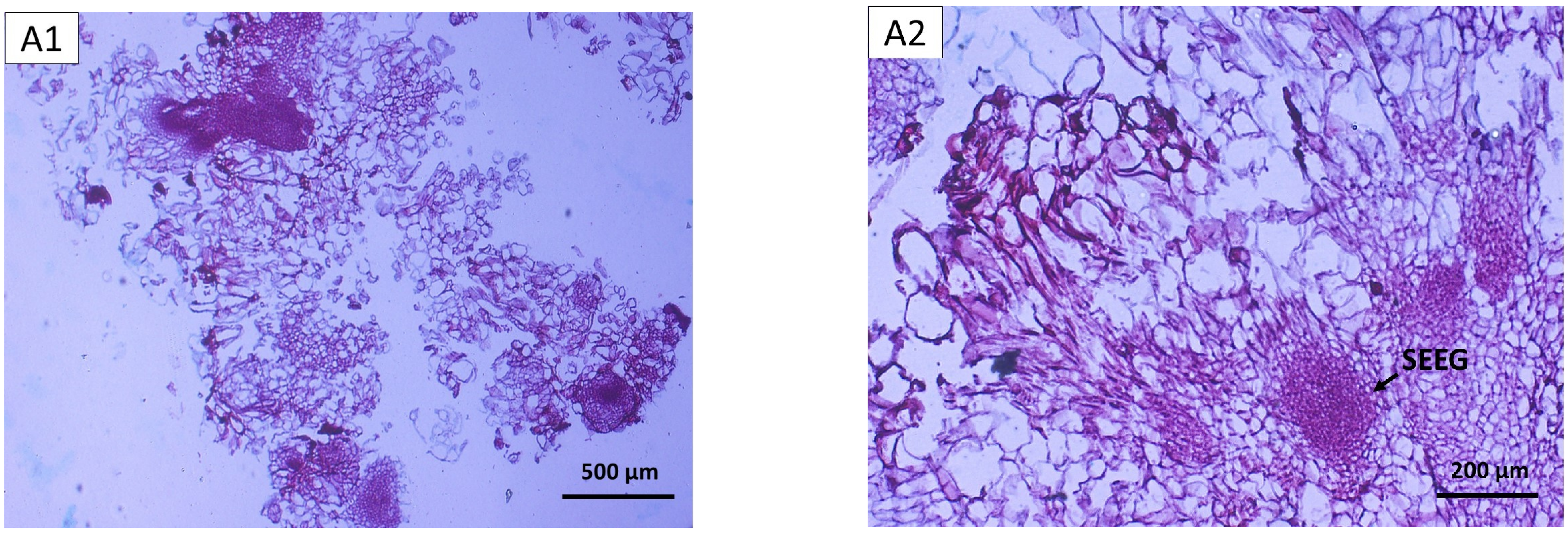

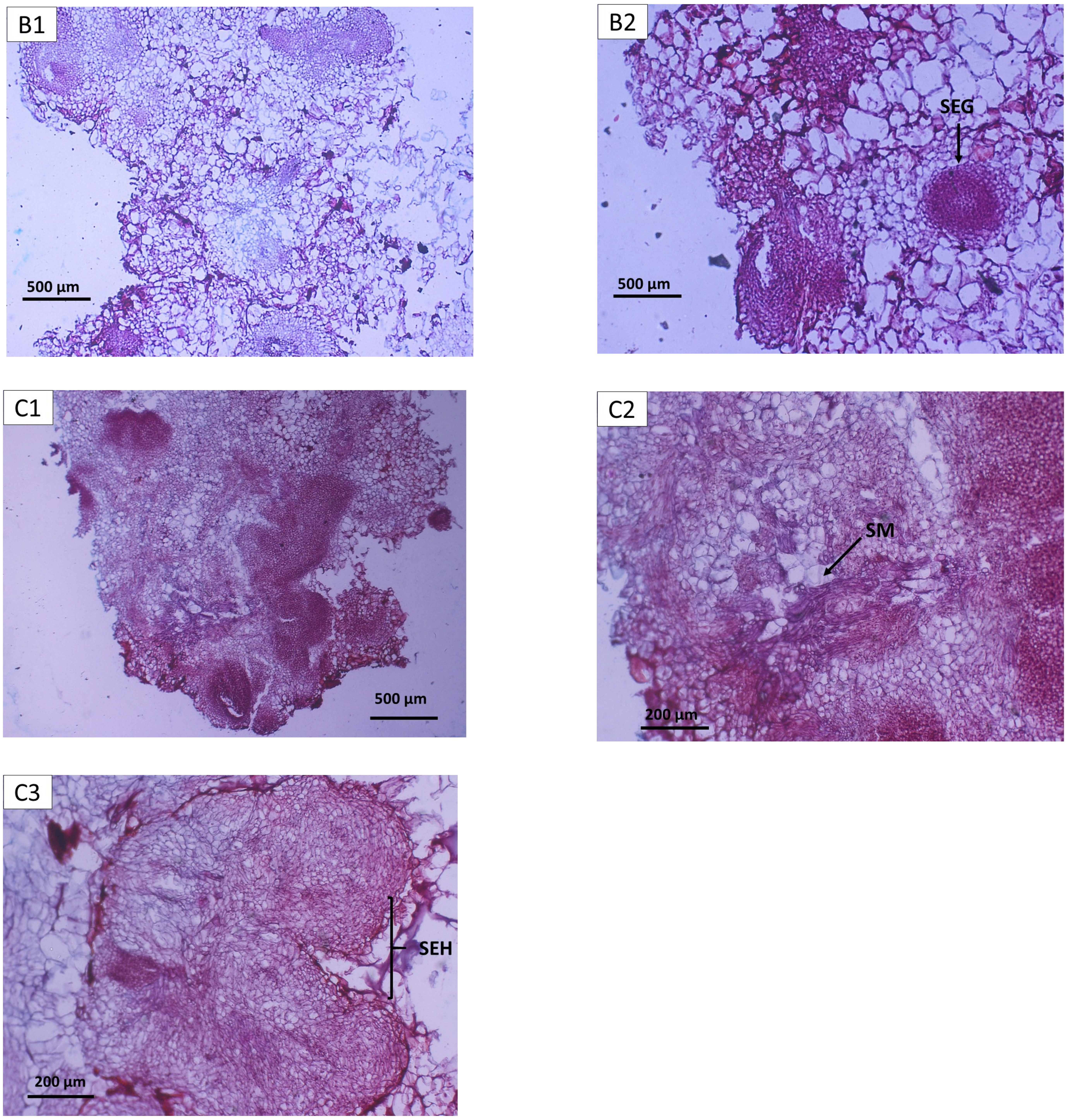

Fig.5. Cross-section of *Sonchus arvensis* L. callus under dolomite treatment

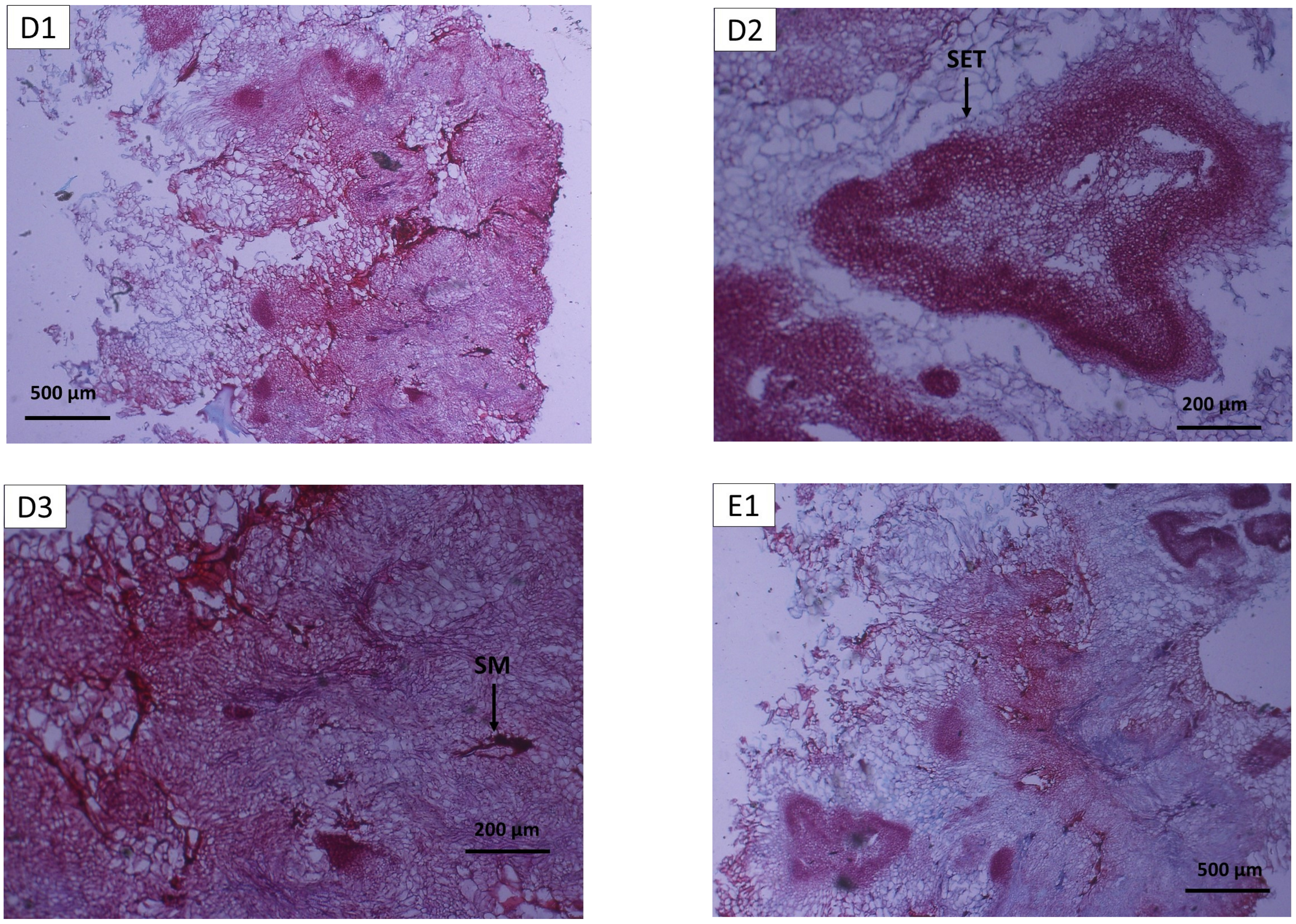

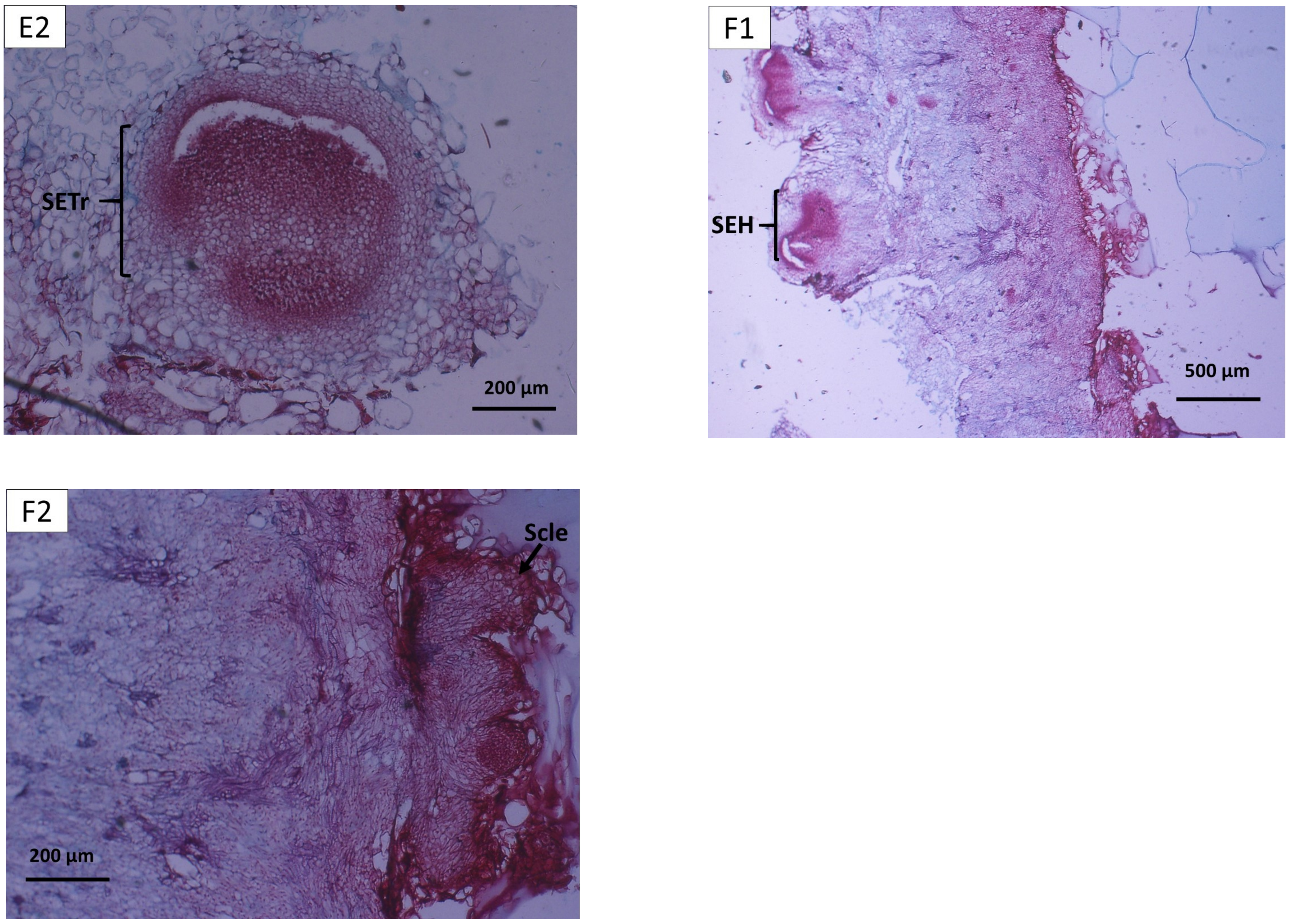

Fig.6. Chromatogram of ethanolic extracts of *Sonchus arvensis* L. callus in different dolomite concentrations

A

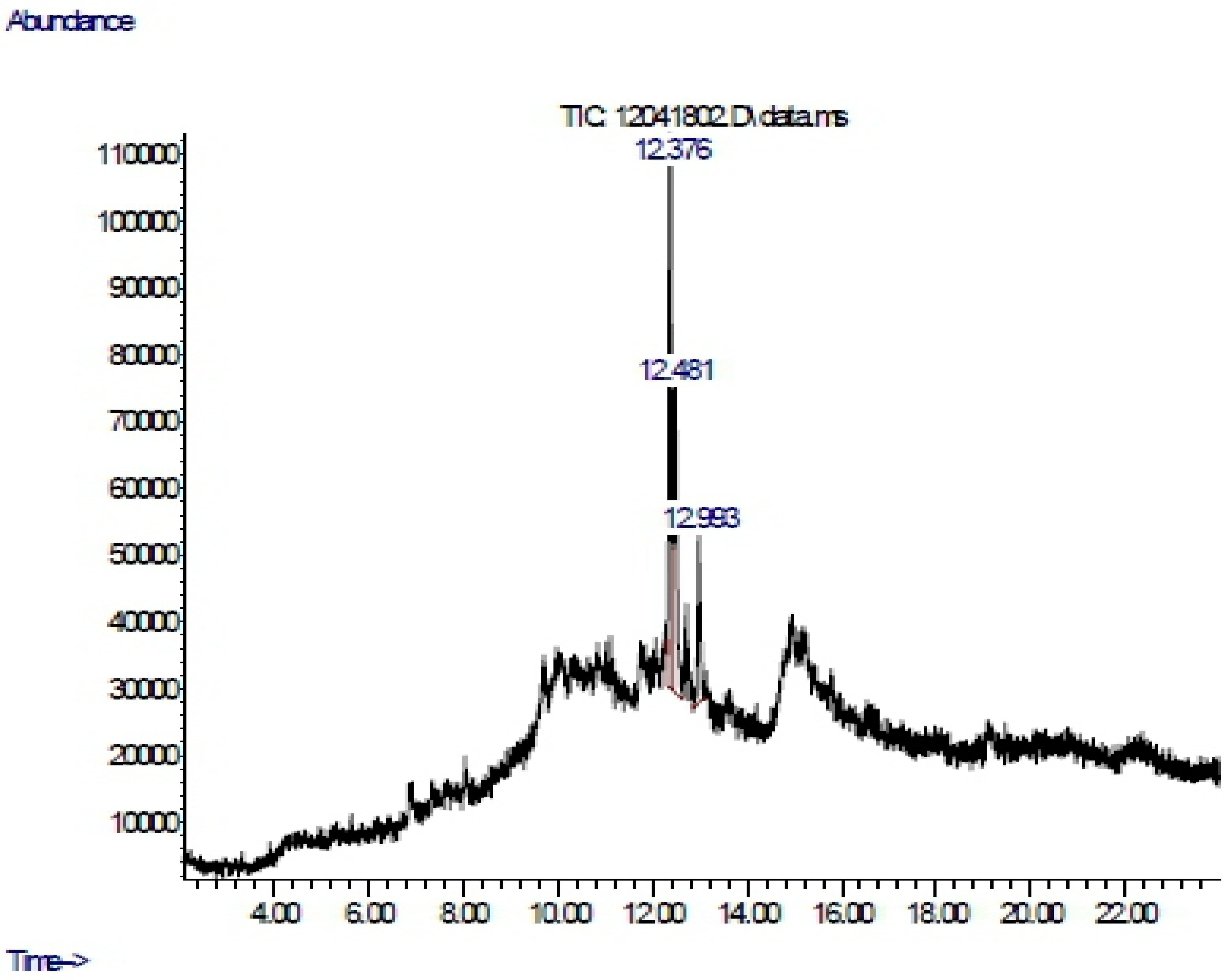

B

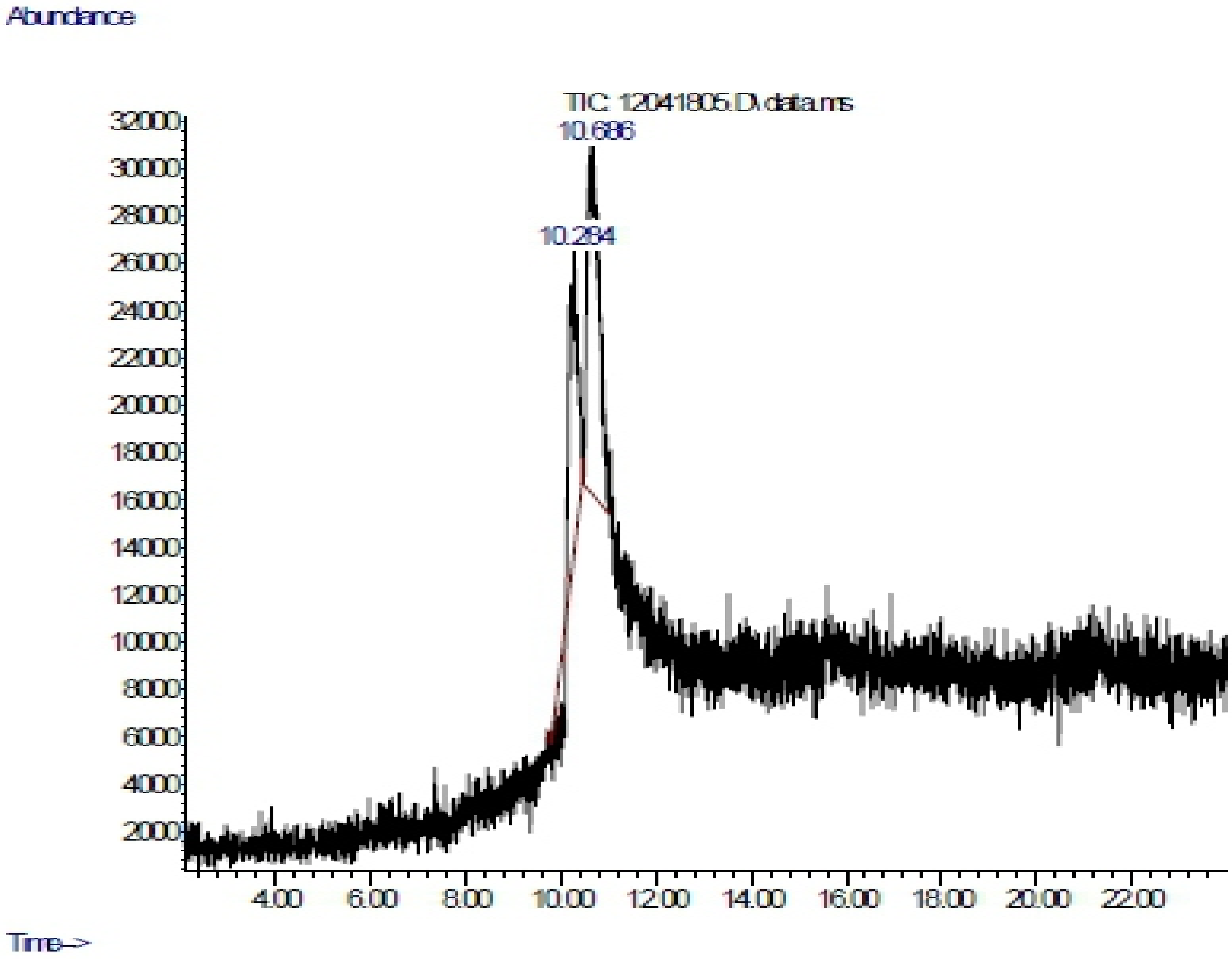
Abundance
TIC: 12041805.D\data.ms
10.686
10.284
32000
30000
28000
26000
24000
22000
20000
18000
16000
14000
12000
10000
8000
6000
4000
2000
4.00
6.00
8.00
10.00
12.00
14.00
16.00
18.00
20.00
22.00
Time-->

C

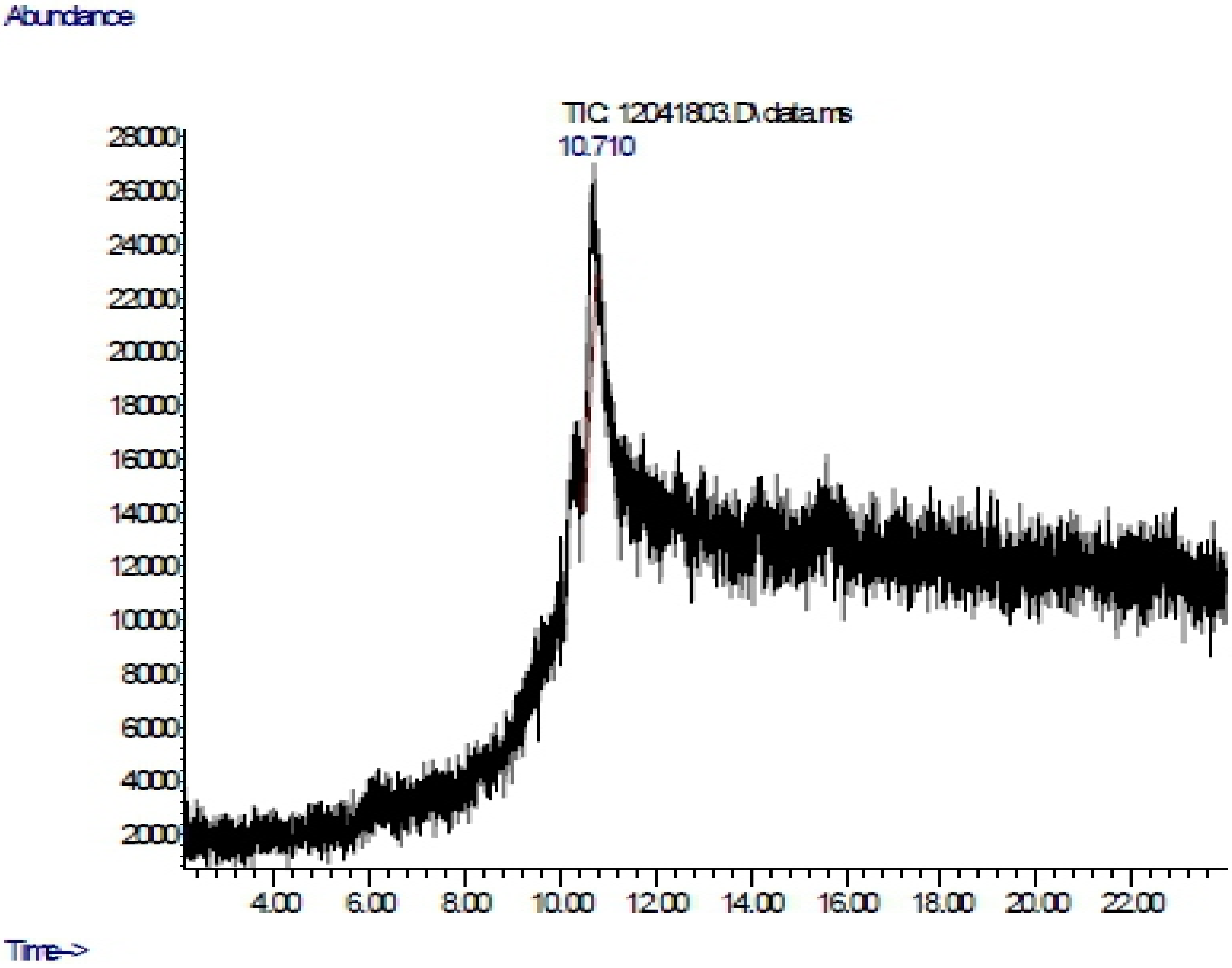
Abundance
TIC: 12041803.D\data.ms
10.710
28000
26000
24000
22000
20000
18000
16000
14000
12000
10000
8000
6000
4000
2000
4.00
6.00
8.00
10.00
12.00
14.00
16.00
18.00
20.00
22.00
Time-->

D

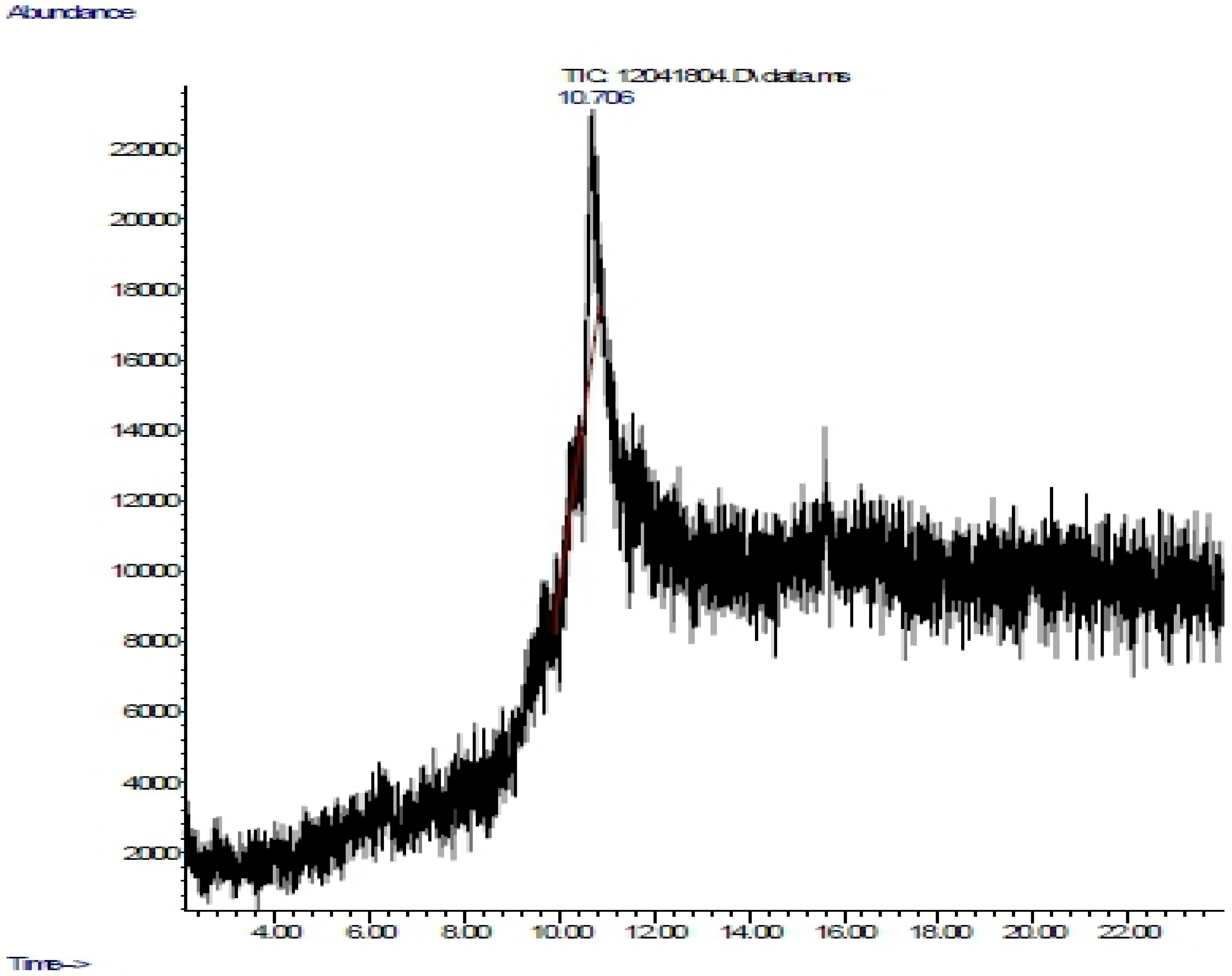

Fig.7. TLC chromatogram of *Sonchus arvensis* L.

I

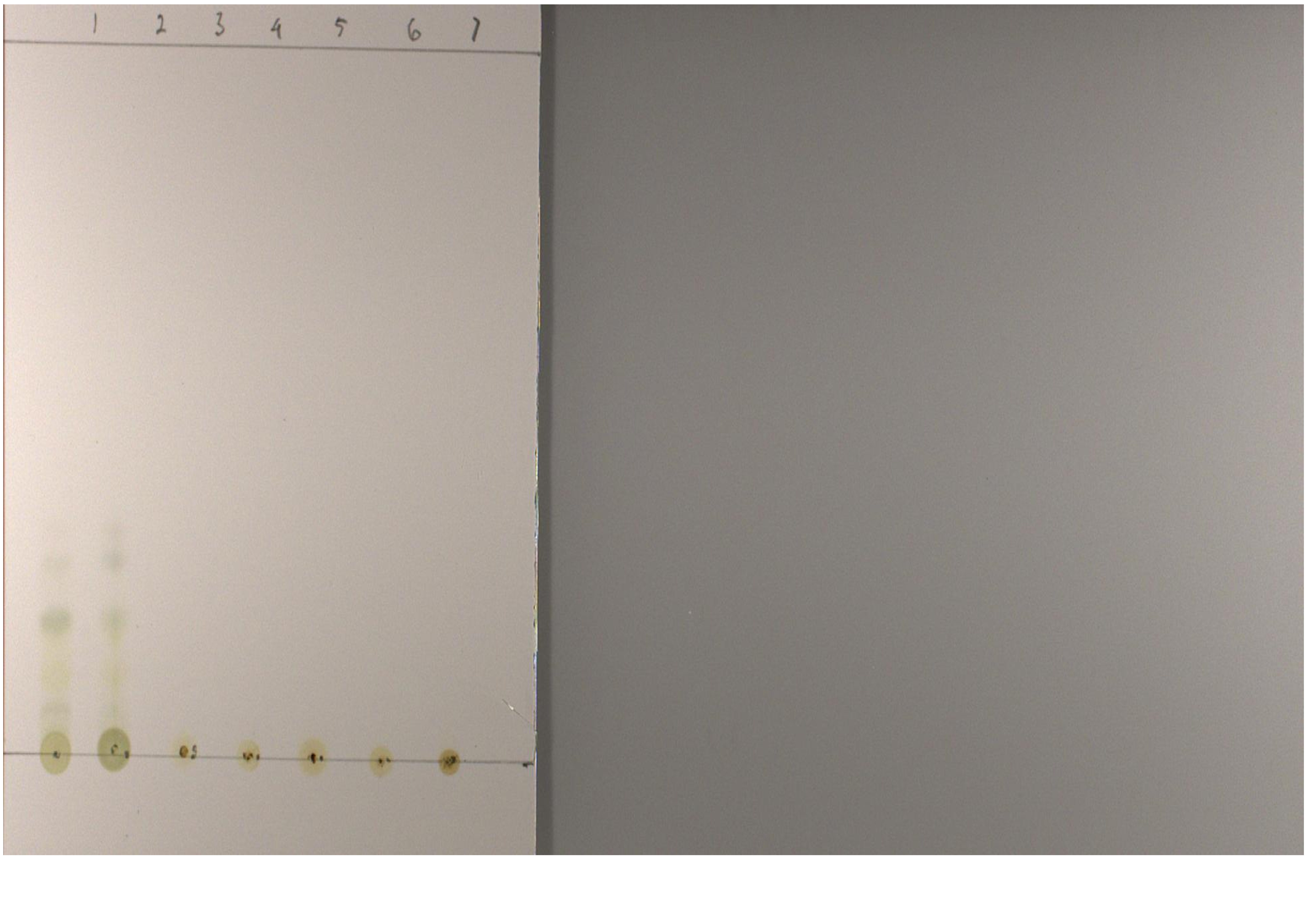

II

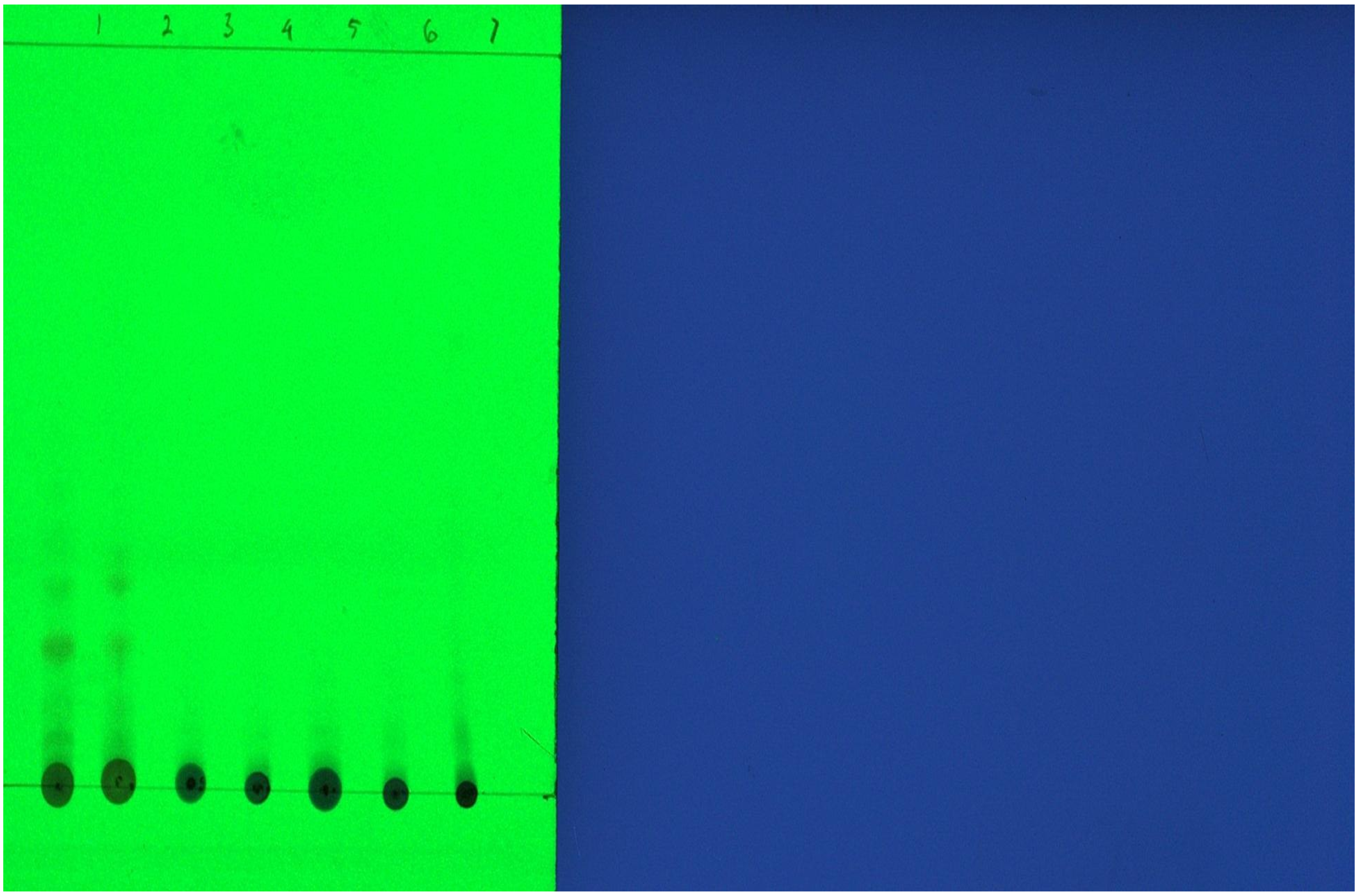

III

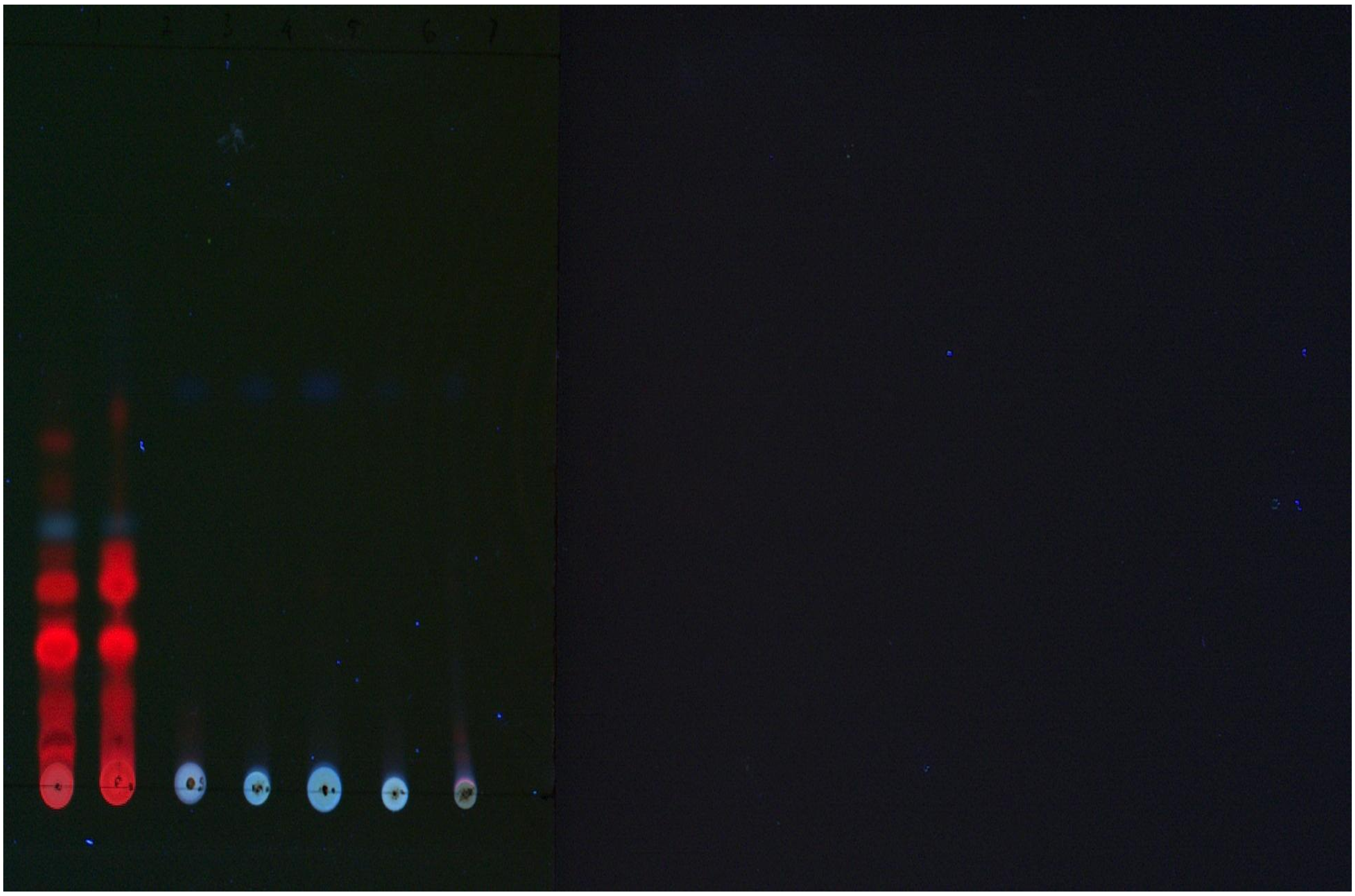

IV

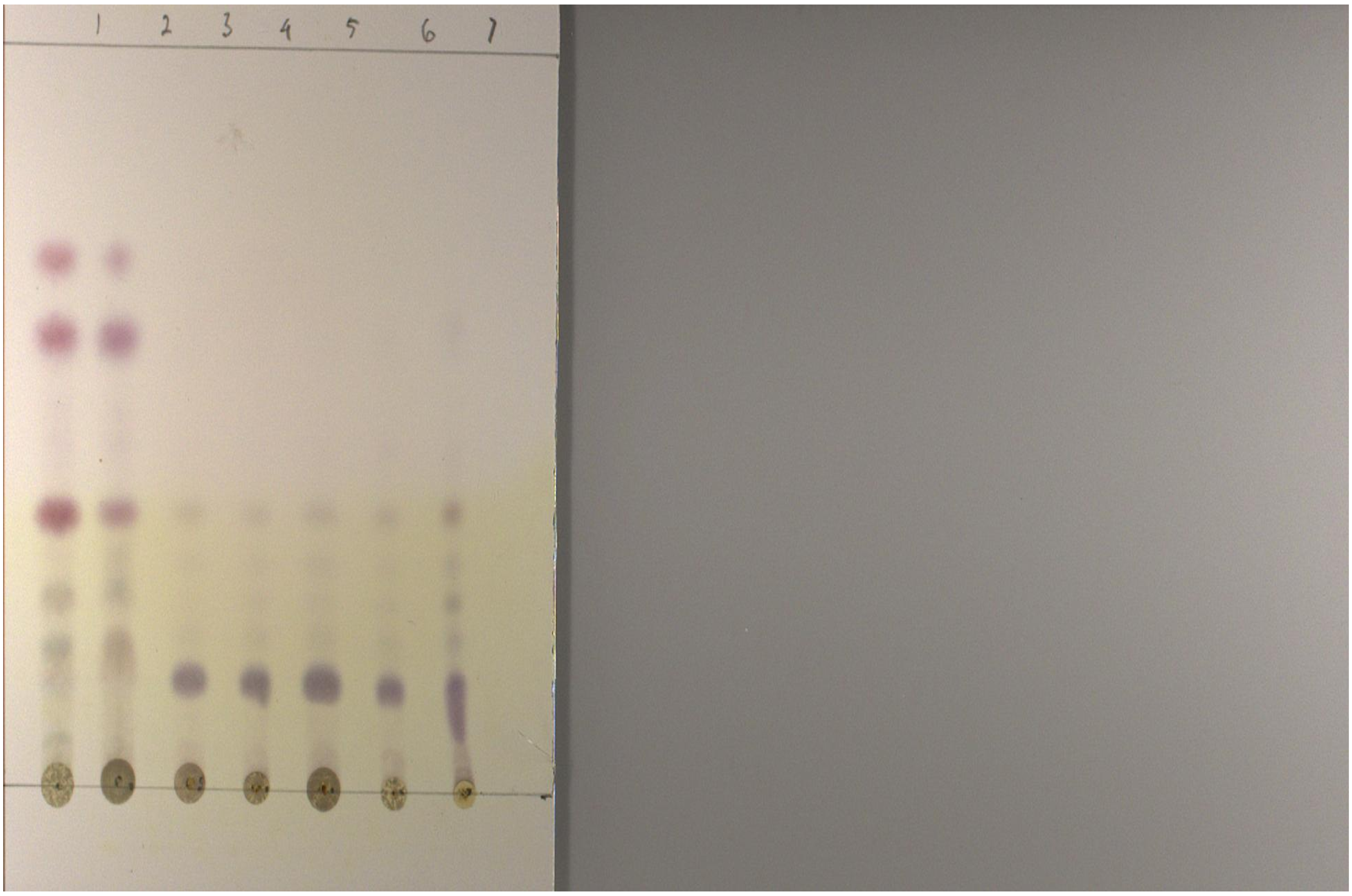

Supplement: S1 Raw images — (PDF) [file pone.0254804.s007.pdf]
